# Multiaction Pt(IV) Complexes: Cytotoxicity in Ovarian Cancer Cell Lines and Mechanistic Studies

**DOI:** 10.1021/acs.inorgchem.4c01586

**Published:** 2024-07-31

**Authors:** Leila Tabrizi, Alan M. Jones, Isolda Romero-Canelon, Andrea Erxleben

**Affiliations:** † School of Biological and Chemical Sciences, University of Galway, Galway H91 TK33, Ireland; ‡ School of Chemical Sciences, Dublin City University, Dublin D09W6Y4, Ireland; § School of Pharmacy, University of Birmingham, Birmingham B15 2TT, U.K.; ∥ Department of Chemistry, University of Warwick, Coventry CV4 7AL, U.K.; ⊥ Synthesis and Solid State Pharmaceutical Centre (SSPC), Limerick V94 T9PX, Ireland

## Abstract

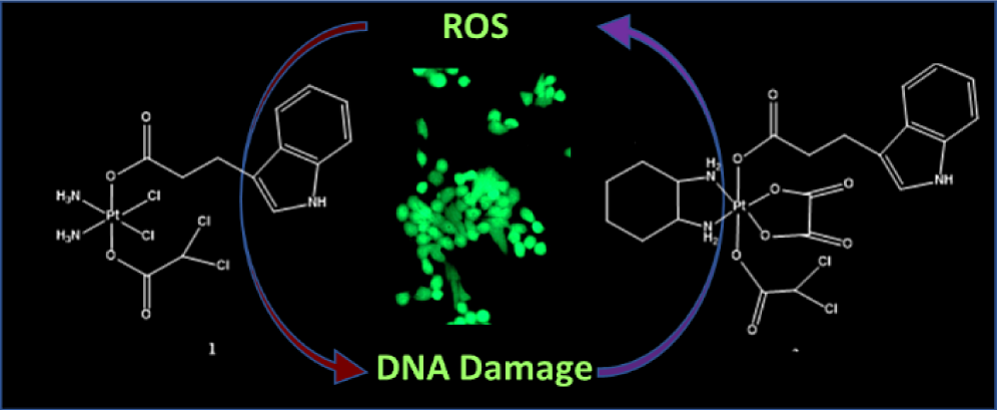

Ovarian cancer has the worst case-to-fatality ratio of all gynecologic malignancies. The main reasons for the high mortality rate are relapse and the development of chemoresistance. In this paper, the cytotoxic activity of two new multiaction platinum(IV) derivatives of cisplatin and oxaliplatin in a panel of ovarian cancer cells is reported. *Cis,cis,trans*-[Pt(NH_3_)_2_Cl_2_(IPA)(DCA)] (**1**) and *trans*-[Pt(DACH)(OX)(IPA)(DCA)] (**2**) (IPA = indole-3-propionic acid, DCA = dichloroacetate, DACH = *1R,2R*-1,2-diaminocyclohexane, OX = oxalate) were synthesized and characterized by elemental analysis, ESI-MS, FT-IR, and ^1^H, ^13^C, and^195^Pt NMR spectroscopy. The biological activity was evaluated in A2780, PEA1, PEA2, SKOV3, SW626, and OVCAR3 cells. Both complexes are potent cytotoxins. Remarkably, complex **2** is 14 times more active in OVCAR3 cells than cisplatin and is able to overcome cisplatin resistance in PEA2 and A2780cis cells, which are models of post-treatment patient-developed and laboratory-induced resistance. This complex also shows activity in 3D cancer models of the A2780 cells. Mechanistic studies revealed that the complexes induce apoptosis via DNA damage and ROS generation.

## Abstract 2

Two new Pt(IV) prodrugs for cisplatin and oxaliplatin are reported that induce apoptosis via DNA damage and ROS production. The oxaliplatin derivative is a promising candidate for the treatment of recurrent cisplatin-resistant ovarian cancer.

## Introduction

Ovarian cancer is the seventh most frequent cancer type in women and the eighth most frequent cause of death from cancer.^1^ According to Globocan, 314,000 women are diagnosed, and 207,000 women die from the disease every year. There are five histological subtypes of ovarian cancer with epithelial tumors being the most common one.^2^ The standard treatment for newly diagnosed ovarian carcinoma is surgery, followed by Pt-based chemotherapy (cisplatin or carboplatin, usually in combination with docetaxel or paclitaxel). The third FDA-approved Pt complex, oxaliplatin, is approved for the treatment of metastatic colorectal cancer, but has also been shown to be active in ovarian cancer cell lines.^3^

The high mortality rate of ovarian cancer is due to late diagnosis, often at an advanced stage, and frequent relapse into cisplatin-resistant forms.^2,4^ Almost 90% of ovarian cancer-related deaths are due to inherent or acquired multidrug resistance.^5^ Consequently, there is an urgent need for more effective chemotherapeutic drugs that can overcome cisplatin or in fact multidrug resistance.

In the search for new anticancer metallodrugs, Pt(IV) complexes play a dominant role.^6^ As Pt(IV) complexes are inert and require activation by reduction, they are potentially less toxic and have fewer adverse effects than Pt(II) complexes. Their octahedral coordination geometry also offers the opportunity for the design of multiaction cancer drugs by attaching bioactive ligands to the axial positions of the cisplatin-, carboplatin-, or oxaliplatin-scaffold.^7−10^ On reduction by biological reducing agents such as glutathione, the active Pt(II) drug and the axial ligands are released and can act synergistically.

We have recently reported Pt(IV) derivatives of cisplatin and oxaliplatin with a biologically active axial indole-3-propionic acid ligand that kill cancer cells through a dual-action mechanism involving DNA damage and the generation of oxidative stress.^11,12^ The most potent complex, *cis,cis,trans*-[Pt(NH_3_)_2_Cl_2_(IPA)(OH)] (IPA = indole-3-propionate) was up to four times more effective than cisplatin and was able to overcome cisplatin resistance in cisplatin-resistant ovarian adenocarcinoma C13* cells. In continuation of our work and with the aim of enhancing the potency of the newly developed prodrugs, we have now attached a second biologically active ligand, dichloroacetate (DCA), to the cisplatin and oxaliplatin scaffolds.

DCA is an orphan drug that inhibits pyruvate dehydrogenase kinase (PDK). PDK phosphorylates the pyruvate dehydrogenase complex in the mitochondria that links glycolysis to the citric acid cycle. DCA can thus revert the metabolic shift of cancer cells from glucose oxidation to glycolysis (Warburg effect) and is also a known apoptosis sensitizer.^13^ In contrast to most cancer drugs, DCA does not affect normal cells.^14^ Dhar and Lippard synthesized and studied *cis,cis,trans*-[Pt(NH_3_)_2_Cl_2_(DCA)_2_] (Mitaplatin)^15^ and more recently Gandin, Gibson and coworkers reported triple-action Pt(IV) complexes containing DCA and a cyclooxygenase inhibitor (aspirin, ibuprofen) or DCA and a histone deacetylase inhibitor (4-phenylbutyrate, valproate). These complexes showed remarkable activity in 2D and 3D cancer cell cultures.^8,10^ A dual-action Pt(IV) derivative of kiteplatin with axial DCA ligands was found to have a similar in vivo activity in a murine tumor model but a lower toxicity than cisplatin.^16^

Here we report the synthesis and characterization of the multiaction Pt(IV) complexes *cis,cis,trans*-[Pt(NH_3_)_2_Cl_2_(IPA)(DCA)] and *trans*-[Pt(DACH)(OX)(IPA)(DCA) (DACH = *1R,2R*-1,2-diaminocyclohexane, OX = oxalate) and their biological activity in a panel of six ovarian cancer cell lines. Studies of the mode of action are also described.

## Experimental Section

### Materials and Instruments

All chemicals were purchased from commercial sources unless stated otherwise. Cisplatin,^17^ oxoplatin,^18^ oxaliplatin,^19^ the NHS ester of indole-3-propionic acid (IPA),^11^*cis,cis,trans*-[Pt(NH_3_)_2_Cl_2_(IPA)(OH)],^11^ and *trans*-[Pt(DACH)(OX)(IPA)(OH)]^12^ were prepared as previously reported. All NMR data were collected on a Varian 500 AR spectrometer by using a 500 MHz Smart Probe. One dimensional^195^Pt NMR spectra were recorded in DMF with insert D_2_O. K_2_PtCl_6_ in D_2_O was used as an external standard. The data were processed by using MestreNova with a 300 Hz line broadening and backward linear prediction. Mass spectra were measured using a Waters LCT Premiere XE with electron spray ionization and a time-of-flight mass analyzer. Elemental analysis (C, H, and N) was performed by an Exeter Analytical CE-440. FT-IR spectra were recorded on a PerkinElmer FT-IR spectrometer fitted with an ATR accessory. The high-performance liquid chromatography (HPLC) studies were carried out with an Agilent 1200 series DAD analytical HPLC instrument. UV/vis spectra were recorded on a Varian Cary 50 Scan spectrophotometer. A dynamic reaction cell ICP-MS (ELAN DRCe, PerkinElmer, Waltham, USA), equipped with a flow injection autosampler (FIAS 93 plus) was used for platinum determination.^20−23^ Instrumental operating conditions were the following: ICP RF Power 1150 W: plasma gas flow 15 L min^–1^, auxiliary gas flow 1 L min^–1^, nebulizer gas flow 0.93 L min^–1^, observed isotopes, ^195^Pt, and^196^Pt. Calibration standard solutions were prepared from a single element standard (Inorganic Ventures, 1000 μg mL^–1^) prepared in Milli-Q water (18.3 mΩ) (Millipore, Bedford, USA) with 1% HNO_3_ (Trace Metal grade, 67–69%, Fisher, UK). Indium (^115^In) was used as an internal standard to account for instrumental drift and matrix effects.

Biological reagents including propidium iodide (94%), RNase, 2′,7’-dichlorofluorescein diacetate (DCFH-DA), *tert*-butyl hydroperoxide (TBHP), hydrogen peroxide, 3-(4,5-dimethylthiazol-2-yl)-2,5-diphenyltetrazolium bromide (MTT), Annexin V-FITC, and Rhodamine-123 were purchased from Sigma-Aldrich and Fisher Scientific. For the biological assays, 96-well plates were read using a FLUOStar Omega microplate reader, while flow cytometry analysis was done using Beckman Coulter Cytoflex, and microscopy images were obtained with an EVOS PL system.

### Synthesis and Characterization of the Pt(IV) Complexes

#### Synthesis of *Cis,cis,trans*-[Pt(NH_3_)_2_Cl_2_(IPA)(DCA)] (1)

Dichloroacetic anhydride (318 mg, 1.326 mmol, 5 equiv) was added to *cis,cis,trans*-[Pt(NH_3_)_2_Cl_2_(IPA)OH] (133.9 mg, 0.265 mmol) in 5 mL of DMF. The reaction mixture was stirred for 24 h at 50 °C. The solution was centrifuged to remove the unreacted Pt complex. To remove the solvent, water was added and the solution was concentrated under reduced pressure. The desired product was precipitated by adding methanol (1 mL), ethyl acetate (3 mL), and diethyl ether (100 mL) to the residue. The solid was collected by centrifugation, washed several times with diethyl ether to remove excess ligands and DMF, and finally dried under vacuum. Yield: (0.265 mmol, 77 mg, 47.5%). Anal. Calc. (%) for C_13_H_17_Cl_4_N_3_O_4_Pt (615.959): C, 25.34; H, 2.78; N, 6.82; Found (%): C, 25.31; H, 2.75; N, 6.84. ESI-MS (negative ion mode): *m*/*z* = 652.01 [M + Cl]^−^. ^1^H NMR (500 MHz, DMSO-*d*_6_): δ 10.76 (s, 1H, NH), 7.46 (d, *J* = 7.3 Hz, 1H, Ar–H), 7.30 (d, *J* = 8.0 Hz, 1H, Ar–H), 7.13 (s, 1H, Ar–H), 7.03 (t, *J* = 7.0 Hz, 1H, Ar–H), 6.94 (t, *J* = 7.2 Hz, 1H, Ar–H), 6.55 (br, 6H, NH_3_), 6.46 (s, 1H, H-DCA), 2.87 (t, *J* = 10 Hz, 2H, CH_2_), 2.61 (t, *J* = 10 Hz 2H, CH_2_). ^13^C NMR (DMSO-*d*_6_): δ = 180.8 (C = O), 170.9 (C = O), 136.6 (C–Ar), 127.4 (C–Ar), 122.7 (C–Ar), 121.3 (C–Ar), 118.5 (C–Ar), 114.0 (C–Ar), 111.8 (C–Ar), 66.28 (CH-DCA), 34.6, 21.5 ppm. ^195^Pt{^1^H} NMR (107.6 MHz, DMF (D_2_O): 1221.69 ppm. IR (cm^–1^): 3394 w, 3185 m (ν_N–H_); 3063 m, 1644 s (ν_C=O_), 1457 w; 1307 m, 1196 m, 1098 m, 947 w, 816, 743(γ_C–H_).

#### Synthesis of *Trans*-[Pt(DACH)(OX)(IPA)(DCA)] (2)

The synthesis was carried out as described for **1**, with *trans*-[Pt(DACH)(OX)(IPA)(OH)] (159.6 mg, 0.265 mmol) instead of *cis,cis,trans*-[Pt(NH_3_)_2_Cl_2_(IPA)OH]. Yield: 0.265 mmol (92.3 mg, 48.9%). Anal. Calc. (%) for C_21_H_25_Cl_2_N_3_O_8_Pt (713.429): C, 35.35; H, 3.53; N, 5.89; Found (%): C, 35.31; H, 3.52; N, 5.92. ESI-MS (negative ion mode): *m*/*z* = 748.88 [M + Cl]^−^. ^1^H NMR (500 MHz, DMSO-*d*_6_): δ 10.82 (s, 1 H, NH), 7.45 (d, *J* = 7.2 Hz, 1 H, Ar–H,), 7.30 (d, *J* = 8.0 Hz, 1 H, Ar–H), 7.09 (s, 1 H, Ar–H), 7.03 (t, *J* = 8.0 Hz, 1 H, Ar–H), 6.94 (t, *J* = 8.0 Hz, 1 H, Ar–H), 6.54 (s, 1 H, H-DCA), 5.82 (s, 4 H, NH_2_), 2.85 (t, *J* = 8.0 Hz, 2 H, CH_2_), 2.64 (t, *J* = 8.0 Hz, 2 H, CH_2_), 2.10–1.05 (m, 10 H, H-DACH). ^13^C NMR (DMSO-*d*_6_): δ = 180.7 (C = O), 169.9 (C = O), 164.6 (C–Ar), 163.9 (C–Ar), 127.3 (C–Ar), 122.6 (C–Ar), 121.3 (C–Ar), 118.5 (C–Ar), 113.7 (C–Ar), 111.8 (C–Ar), 71.8, 66.28 (CH-DCA), 61.6, 61.1, 36.6, 34.5, 31.2, 24.02, 23.9, 21.4, 15.6 ppm. ^195^Pt{^1^H} NMR (107.6 MHz, DMF (D_2_O): 1624.95 ppm. IR (cm^–1^): 3182 w (ν_N–H_), 2972 m; 1638 s (ν_C=O_), 1459 w; 1314 s, 1208 m, 1108 w, 1065 w, 1023 w, 951 w, 809 m, 745 m, 711 m, 662 m.

### Determination of the Lipophilicity Parameters

The log *P*_*o/w*_ values of the Pt(IV) compounds were determined by the shake flask method.^24^ The respective Pt(IV) complex was dissolved in 0.9% (w/v) ultrapure NaCl (presaturated with n-octanol). The solution was sonicated and filtered to remove the undissolved Pt(IV) complex. The initial Pt concentration was determined by ICP-MS. Then the Pt(IV) solution was added to an equal volume of n-octanol (presaturated with 0.9% (w/v) NaCl ultrapure water). The heterogeneous mixture was shaken vigorously for 30 min before centrifuging at 4000 rpm for 30 min to achieve phase separation. The Pt concentration in the aqueous phase was determined again by ICP-MS. The logarithm of the ratio of the Pt concentrations in the organic and aqueous phases was calculated to determine the logP values. All experiments were performed in duplicate.

### Cyclic Voltammetry (CV)

CVs were performed using an Autolab PGSTAT100N potentiostat and processed with Nova 2.1 software, and graphical representations were created and analyzed using SigmaPlot 14.5. Measurements for complexes **1** and **2** (1.0 mM, CH_3_CN containing tetrabutylammonium hexafluorophosphate (0.1 M) as supporting electrolyte) were performed and voltammograms were scanned (+2.0 V to −2.0 V). Voltammograms were also scanned from +0.8 V to −0.8 V to mimic biological redox potentials. In a typical electrochemical experimental setup, a three-electrode system was used: a glassy carbon electrode as the working electrode, Ag/Ag^+^ as the pseudo reference electrode (+400 mV vs Fc^+^/Fc couple), and platinum wire as the counter electrode. For each experiment, the CV was performed at a ν = 100 mVs^–1^.

### Chemical Stability in PBS and Biologically Relevant Medium

To test the stability of the Pt(IV) complexes, **1** and **2** were dissolved in DMEM (Dulbecco’s Modified Eagle’s Medium, high glucose)/1% DMSO solution and freshly prepared PBS buffer/1% DMSO solution. The solutions were stored for 72 h at 37 °C and analyzed by HPLC (Phenomenex Luna C18 column, 5 μm, 100 Å, 250 mm × 4.60 mm i.d.; 0.5 mL/min flow rate, 250 nm UV detection) and UV–vis spectroscopy. The mobile phase was 70:30 acetonitrile (0.1% trifluoroacetic acid):water (0.1% trifluoroacetic acid).

### Reduction Reaction Studied by HPLC

The reduction of the complexes **1** and **2** (10 mM) with sodium ascorbate (10 equiv) or glutathione (10 eq. and 500 eq.) was followed by HPLC using a Phenomenex Luna C_18_ column (5 μm, 100 Å, 250 mm × 4.60 mm i.d.; 0.8 mL/min flow rate; 250 and 280 nm UV detection at room temperature). The mobile phase was 70:30 acetonitrile (0.1% trifluoroacetic acid)/water (0.1% trifluoroacetic acid). The respective complex was dissolved in DMF (0.5 mL), added to a 5 mM solution of sodium ascorbate in 2 mM 4-(2-hydroxyethyl)piperazine-1-ethanesulfonic acid (HEPES) buffer (pH 7), and diluted with acetonitrile to a final concentration of 0.5 mM. The reaction was monitored at 37 °C until completion.

### Cell Culture

All cancer cell lines used in this work were obtained from the European Collection of Cell Cultures (ECACC). They include A2780, SKOV3, PEA1, PEA2, SW626, and OVCAR3, all of which are of ovarian origin. Cells were routinely grown in Roswell Park Memorial Institute medium (RPMI-1640) that included 10% fetal calf serum, 1% glutamine (2 mM), and 1% of the antibiotic mixture penicillin/streptomycin. They were grown as adherent monolayers and kept at 37 °C in a 5% CO_2_ humidified atmosphere, regularly passaged with trypsin-EDTA.

### Cellular Accumulation

A2780 and PEA1 cells were seeded at a density of 2 × 10^5^ cells/mL into 25 cm^2^ flasks. After overnight incubation, the medium was replaced with fresh medium, and the cells were treated with 5 μM concentrations of complex **1** or **2** for 12 h. The cells were washed twice with cold PBS, harvested by trypsinization, and counted using a hemocytometer. The cells were treated with 1 mL of highly pure nitric acid and 2 mL hydrochloric acid, left in a fume cupboard to predigest overnight, and transferred into microwave tubes. The tubes were heated at 165 °C for 20 min to digest the cells. After they were cooled, the samples were diluted with ultrapure water to a final concentration of 1% HNO_3_. The mineralized samples were filtered and the Pt content was quantified by ICP-MS in a class 1000 cleanroom. The calibration curve was obtained using known concentrations of Pt standard solutions (0–50 μM).

### Determination of IC_50_ Values

Ovarian cancer cells were seeded in flat-bottom 96-well plates at a density of 500 cells/well. They were preincubated in drug-free media at 37 °C for 48 h, followed by 24 h of drug exposure time. Pt(IV) complexes **1** and **2** were initially prepared as stock solutions using DMSO to aid dissolution and then diluted with a cell culture medium. For all working solutions used, there was a maximum DMSO concentration that did not exceed 0.5%. Removal of drugs after exposure included a PBS wash before allowing the cells to recover in a drug-free medium for 72 h. Cell viability was then determined using the MTT assay after 4 h of dye exposure in the dark. Absorbance measurements of the solubilized dye allowed the determination of viable treated cells compared to untreated controls. IC_50_ values (concentrations which caused 50% of cell growth inhibition) were determined as duplicates of triplicates in two independent sets of experiments, and their standard deviations were calculated. These experiments included cisplatin as positive controls.

### Induction of Apoptosis

The induction of the cell death mechanism was investigated using flow cytometry with Annexin V-FITC and propidium iodide (PI). A2780 ovarian cancer cells were seeded in 6-well plates and allowed to attach for 24 h. Following attachment, cells were treated with Pt(IV) complexes **1** and **2** using equipotent concentrations equal to 1X IC_50_ values. After 24 h of drug exposure time, drugs were removed by suction, and cells were washed with PBS and detached using trypsin. Single-cell suspensions were stained using PI/Annexin V-FITC in a buffer. This experiment included negative untreated controls and positive control cells induced with staurosporine (1 μg/mL). Cells for apoptosis studies were used with no previous fixing procedure to avoid nonspecific binding of the annexin V-FITC conjugate. These experiments were carried out in triplicates, full numerical data and statistical analysis can be found in the Supporting Information (Table S3).

### Cell Cycle Analysis

A million A2780 ovarian carcinoma cells were seeded similarly to the protocol discussed in the previous section and treated with equipotent concentrations (1X IC_50_ values) of complexes **1** and **2**. Following 24 h of drug exposure, drugs were removed by suction, and cells were washed with PBS and detached using trypsin-EDTA. Single-cell solutions were obtained and centrifuged to render cell pellets that were fixed for 2 h using ice-cold ethanol. Following fixation, cell pellets were stained by resuspending them in PBS containing propidium iodide (PI) and RNase A. Samples were analyzed by flow cytometry exploiting PI-bound DNA maximum excitation at 536 nm, and its emission at 617 nm. Data were processed by using Flowjo software. These experiments used untreated cells as negative controls. These experiments were carried out in triplicates, full numerical data and statistical analysis can be found in the Supporting Information (Table S4).

### Wound Healing Assay

Twenty-four well plates were inoculated with A2780 ovarian cancer cells (60,000 cells/well) and allowed to reach 90% confluence. Following attachment, two “wounds” were created in each well using a pipet tip, and cells were treated with equipotent concentrations of complexes **1** and **2** (1X IC_50_ values). After 24 h of exposure, the Pt(IV) drugs were removed by suction, cells were washed with PBS and fixed with a solution of 2% paraformaldehyde in PBS. Cells were visualized using a 4x transmission microscope. The width of the wound was measured using ImageJ, and all statistical analyses can be found in the Supporting Information (Table S2).

### Induction of Reactive Oxygen Species (ROS)

A2780 ovarian carcinoma cells were seeded in 96-well black plates using 10,000 cells per well. Cells were allowed to attach for 24 h before adding equipotent concentrations of complexes **1** and **2** (1X and 3X IC_50_ values). Working solutions were obtained as described for the cytotoxicity assays. After 24 h of drug exposure, supernatants were removed by suction and the plates were washed with PBS. To each well, 100 μL of a 50 μM solution of 2′,7’–dichlorofluorescein diacetate (DCFH-DA) was added and the plates were incubated with the dye in the dark for 2 h at 37 °C. Once cells were stained, supernatants were removed by suction and wells were washed with PBS before adding ROS inducers as positive controls. Hydrogen peroxide was used at 1 mM and *tert*-butyl hydroperoxide (TBHP) at 500 μM. ROS induction by positive controls was allowed for 2 h in the dark at 37 °C. Fluorescence readings were obtained with an excitation at 485 nm and an emission at 530 nm. This experiment included negative untreated controls, controls only treated with the metal complexes (to discard autofluorescence), untreated cells with hydrogen peroxide or TBHP, and complex-treated cells with the ROS inducers. For the fluorescence microscopy experiments, cells were seeded using 8-well microscopy chambers with 5000 cells/well. Drug treatment and staining were similarly carried out, and readings were obtained using an EVOS FL microscope. Full numerical data and statistical analysis can be found in the Supporting Information (Table S5).

### Evaluation of Mitochondrial Function

A2780 ovarian cancer cells were seeded in 8-well microscopy chambers with 5000 cells/well and allowed to attach for 24 h. Following attachment, cells were treated with equipotent concentrations of complexes **1** and **2** (1X and 3X IC_50_ values) using solutions as described earlier. After 24 h of exposure time, all drugs were removed by suction, cells were washed with PBS, stained using DAPI/Rh-123 in buffer, and readings were obtained using an EVOS FL microscope. These experiments used untreated cells as negative controls.

### Evaluation of Anticancer Activity in 3D Spheroid Models of A2780 Cells

Briefly, 5000 A2780 cells were seeded per well in U-bottom, 96-well plates with cell-repellent surfaces. The cells were preincubated in drug-free media at 37 °C for 7 days before adding Pt(IV) complexes **1** and **2** in equipotent concentrations. Stock solutions using DMSO to aid dissolution and corresponding working dilutions were carried out in an RPMI-1640. The drug exposure period was 24 h. After this, supernatants were removed by suction, and each spheroid was washed with PBS before being fixed with a solution of 2% paraformaldehyde in PBS. Spheroids were visualized using a 2x transmission microscope. All numerical data and statistical analysis can be found in the Supporting Information (Table S1).

### Statistical Analysis

In all cases, independent two-sample *t* tests with unequal variances, Welch’s tests, were carried out to establish statistical significance of the variations (*p* < 0.01 for **, and *p* < 0.05 for *).

## Results and Discussion

### Synthesis and Characterization of the Pt(IV) Complexes

**Figure 1:**
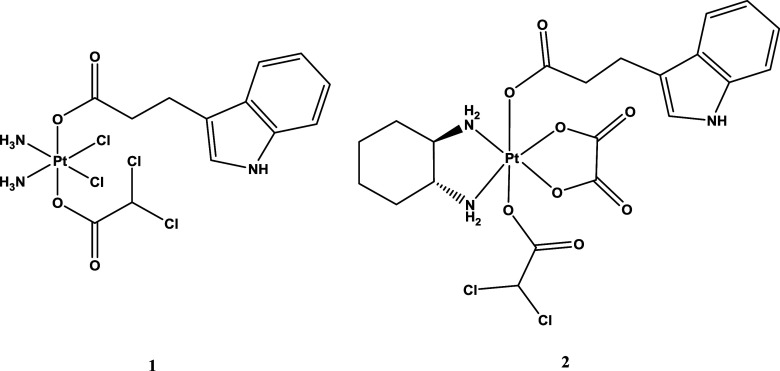
Structures of complexes **1** and **2**.

Compounds **1** and **2** (Figure 1) were obtained by reacting *cis,cis,trans*-[Pt(NH_3_)_2_Cl_2_(IPA)(OH)]^11^ and *trans*-[Pt(DACH)(OX)(IPA)(OH)]^12^ with an excess of dichloroacetic anhydride in DMF (Scheme S1). The purity of the synthesized complexes was confirmed by elemental analysis and high-performance liquid chromatography (HPLC). **1** and **2** were characterized by ESI-MS, IR, and multinuclear (^1^H, ^13^C, ^195^Pt) NMR spectroscopy (Figures S1–S10). The molecular ion peaks in the ESI mass spectra at 652.01 [M + Cl]^−^ (**1**) and 748.88 [M + Cl]^−^ (**2**) show typical platinum isotope patterns that are in good agreement with the simulated patterns. The IR spectra display characteristic C = O stretching vibrations around 1644 and 1638 cm^–1^ for **1** and **2**, respectively. The antisymmetric N–H stretching bands of the amine ligands of the complexes are observed around 3180–3185 cm^–1^. The ^1^H and ^13^C NMR spectra of **1** and **2** agree with the suggested structures. The ^1^H NMR spectrum of complex **1** shows the typical broad signal of the NH_3_ protons at 6.55 ppm. The methinic proton of the DCA ligand in complexes **1** and **2** gives a singlet at 6.46 and 6.54 ppm, respectively. The^195^Pt NMR resonance at 1221.7 ppm for complex **1** and 1606.3 ppm for complex **2** is in good agreement with those reported for similar Pt(IV) derivatives of cisplatin and oxaliplatin with two axial carboxylato ligands.^11,12^

### Anticancer Activity Investigations

The cytotoxicity of the Pt(IV) complexes was first screened in a panel of six ovarian cancer cell lines using the standard MTT assay and 2D cell cultures. The IC_50_ values are presented in Table 1. Both complexes are highly effective in A2780 cells. Complex **2** is an order of magnitude more active than cisplatin in the OVCAR3 cell line (IC_50_ for **2** and cisplatin 3.81 and 54.3 μM, respectively). However, in contrast to cisplatin or oxaliplatin, none of the Pt(IV) complexes is active in SKOV3 cells. Interestingly, there is a great cytotoxicity difference between complexes **1** and **2** in the PEA1 cell line (IC_50_ > 100 μM (**1**) and 4.08 μM (**2**)) and the same observation is made in the PEA2 cell line (IC_50_ > 100 μM (**1**) and 4.45 μM (**2**)). These two lines are related, as they are both derived from the same patient. PEA2 was collected on relapse after treatment with cisplatin and prednimustine. Both lines are estrogen receptor positive. The activity of **2** in PEA1 cells is comparable to that of cisplatin, but while cisplatin exerts a 4-fold reduced cytotoxicity in PEA2 cells, complex **2** maintains its activity (IC_50_ = 4.08 and 4.45 μM in PEA1 and PEA2 cells, respectively). Similar to the behavior observed by cisplatin, oxaliplatin also loses potency with its IC_50_ increasing from 12.6 μM in PEA1 to 43.4 μM in PEA2.

**Table 1:** IC_50_ Values Determined for Complexes **1** and **2** in a Panel of Ovarian Cancer Cell Lines Including: A2780, PEA1, PEA2, SKOV3, SW626, and OVCAR3^a^

	A2780	PEA1	PEA2	SKOV3	SW626	OVCAR3
complex **1**	2.18 ± 0.6	>100	>100	>100	30.1 ± 0.9	19.5 ± 0.8
complex **2**	0.69 ± 0.04	4.08 ± 0.09	4.45 ± 0.05	>100	5.6 ± 0.7	3.81 ± 0.08
cisplatin	1.2 ± 0.3	6.5 ± 0.2	26.7 ± 0.3	11.5 ± 0.7	7.14 ± 0.08	54.3 ± 0.1
oxaliplatin	2.8 ± 0.2	12.6 ± 0.3	43.4 ± 0.7	14.3 ± 0.5	9.2 ± 0.6	68.1 ± 0.8

a All experiments included 48 h of pre-incubation time, 24 h of drug exposure, and 72 h of recovery time in drug-free media, they were run as duplicates of triplicates in independent experiments, and their standard deviations were calculated. Cisplatin and Oxaliplatin were used as positive controls.

As a result of these promising results in the resistant cell line PEA2, in which the lack of response to cisplatin emerged post-patient treatment, we tested whether the Pt(IV) complexes could also overcome cisplatin resistance in A2780cis cells. In this case, resistance has been generated under laboratory conditions by prolonged exposure to cisplatin. The resistance factors, calculated as the ratios of the IC_50_ values in the resistant and sensitive cells (Table 2) are 2.20 (complex **1**) and 1.18 (complex **2**). These are 4 and 7 times lower than the resistance factors of cisplatin. In particular, the resistance factor of **2** confirms the absence of cross-resistance with cisplatin. Cisplatin resistance is multifactorial and in the case of A2780 cells, these cellular mechanisms are well established to include reduced cellular accumulation, increased DNA repair, and elevated glutathione levels.^25,26^ The latter can promote prodrug reduction and may thus actually be beneficial for Pt(IV) complexes. It is worth noting that the resistance factor for both clinical platinum drugs is the same, at 8.6. Exploring the activity of complexes in cell lines such as A2780cis and PEA2 allows for investigations into acquired resistance, but comparisons toward SKOV3, SW626, and OVCAR3 have their value in terms of looking into inherent resistance or lack of sensitivity. In particular, SKOV3 is classified as a highly resistant line according to its genetic makeup.^27^ These three nonsensitive cell lines have been reported to include an ambiguous function of TP53 as a driver mutation,^28^ which is consistent with their diminished responses to cisplatin and oxaliplatin. Potency values obtained along these lines for complexes **1** and **2** would grant further research into the role of this oncogene in their mechanism of action.

**Table 2:** IC_50_ Values Determined for Complexes **1** and **2** in the A2780cis-Resistant Cell Line^a^

	A2780	A2780cis	RF
complex **1**	2.18 ± 0.6	4.8 ± 0.3	2.20
complex **2**	0.69 ± 0.04	0.82 ± 0.05	1.18
cisplatin	1.2 ± 0.3	10.4 ± 0.7	8.66
oxaliplatin	2.8 ± 0.2	24.3 ± 0.6	8.67

a The experiments included 48 h of pre-incubation time, 24 h of drug exposure and 72 h of recovery time in drug-free media, they were run as duplicates of triplicates in independent experiments and their standard deviations were calculated. The resistance factors (RF) were calculated as the ratios of the IC_50_ values in the resistant and sensitive cells.

**Figure 2:**
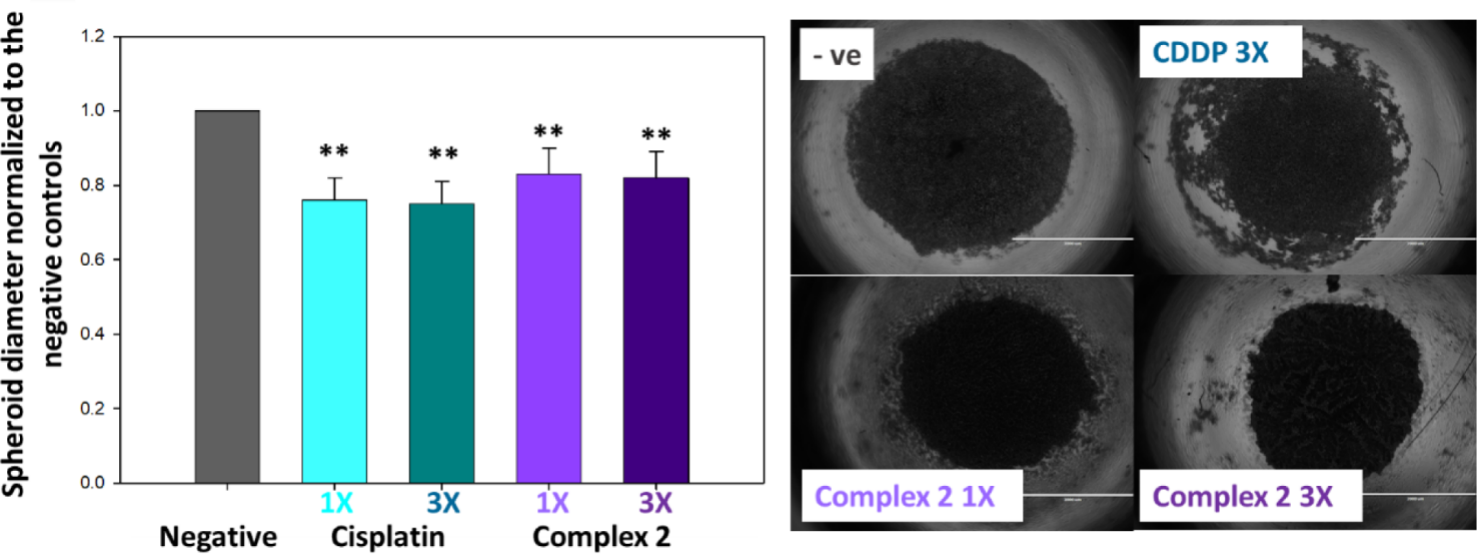
Studies in 3D cellular models. Spheroids were generated using A2780 ovarian cancer cells grown in cell-repellent 96-well plates. The experiments included 24 h of drug exposure time using equipotent concentrations of 1X and 3X IC_50_ values and no recovery. Diameter measurements have been normalized to the negative controls.

While 2D monolayer cultures are commonly used for an initial activity screening, they do not mimic the cellular heterogeneity, metabolic gradients, and cell–cell and cell-matrix interactions in solid tumors. 3D spheroids allow a more predictive cytotoxicity testing. Figure 2 shows the results of exposing 3D spheroids of A2780 cells to complex **2** at concentrations of 1X and 3X IC_50_ values, which is the best-performing compound in the previous screening. Table S1 contains the individual diameter measurements and the normalization to the negative controls. Both concentrations of the Pt(IV) complex cause a statistically significant reduction of the diameter of the spheroids, and similarly to those treated with equipotent concentrations of cisplatin, there is no clear concentration dependence in the activity. A high concentration of cisplatin does cause “cresting” of the spheroids, in which an outer ring of the model is clearly deteriorated by the action of the drug, this effect is not well observed in the spheroids treated with complex **2**. The generation of cresting in spheroids has been observed after treatment with other platinums such as oxaliplatin, in which case the gradient in cellular response has been linked to platinum-bound DNA content across the aerobic-to-hypoxic environment and issues with drug penetration.^29^ Observing a significant size reduction in the spheroids is an encouraging result, particularly taking into account that this experimental setup did not include drug recovery time. In general, it is expected 3D cell models to exhibit stronger chemotherapeutic barriers than 2D cultures, in fact, resistance or nonsensitivity to platinum drugs, as well as, to vincristine, doxorubicin and topotecan have been reported to critically change the capacity of ovarian cells to generate and maintain spheroid structures.^30^

**Figure 3:**
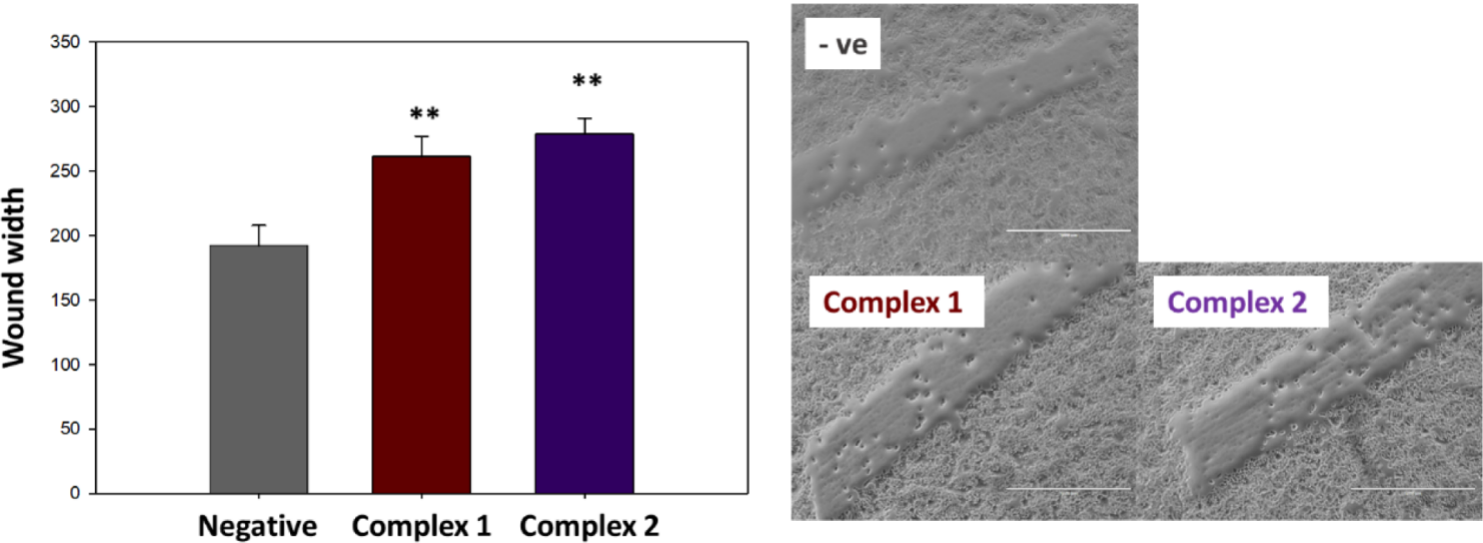
Wound healing assay carried out using A2780 ovarian cancer cells. Wounds were inflicted and then measured after 24 h of drug exposure using equipotent concentration 1X IC_50_.

Since a high number of ovarian cancer-related deaths are due to the development of metastatic disease^31^ we decided to investigate the ability of the Pt(IV) prodrugs to inhibit cell migration using a wound healing assay with A2780 cells. In this case, similarly to the experimental setup followed with the spheroids, the treatment included 24 h of drug exposure but no recovery time. Figure 3 shows that both complex **1** and complex **2** inhibit the cell migration to close the wound in a statistically significant manner. Further information and numerical analysis can be found in Table S2. Complex **2** shows a slightly higher effect than complex **1**, which is consistent with other experiments within this work; nonetheless, both complexes continue to have promising activity. Therefore, we pursued further investigations to understand their mechanism of action at cellular level.

### Explorations on the Mechanism of Action at Cellular Level

**Figure 4:**
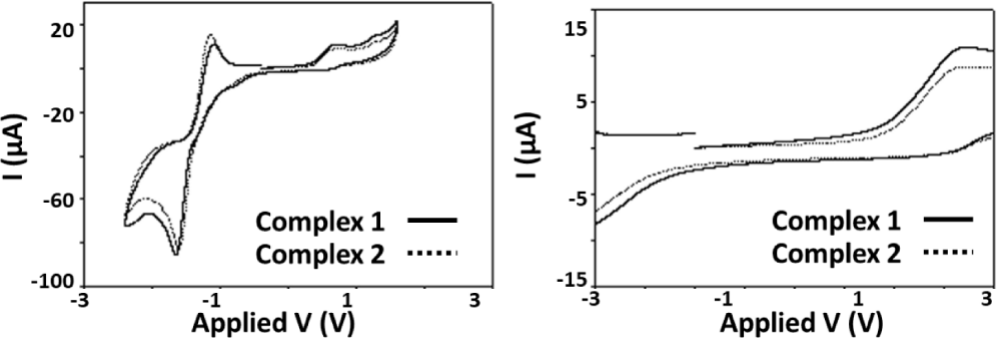
Cyclic voltammetry for complexes **1** and **2** in either a +2.0 V to −2.0 V wide window or a from +0.8 V to −0.8 V to mimic biological redox potentials.

As Pt(IV) complexes require activation by biological reducing agents, we studied the redox activities of **1** and **2** by cyclic voltammetry (Figure 4). For most of the biologically relevant redox windows, the complexes are redox inert. At higher biologically redox-relevant oxidation potentials ∼+650 mV (Fc/Fc+), modest quasi-reversible oxidation behavior is observed. In a wider window, the characteristic oxygen redox couple at −1.25 V (Fc/Fc+) is present.

The reduction potential of a Pt(IV) complex is mainly determined by its axial ligands.^32^ However, it is now well established that there is often no correlation between the (thermodynamic) reduction potential and the reduction kinetics that also depend on the nature of the equatorial ligands and the reducing agent.^33,34^ The reduction of **1** and **2** (10 mM) by sodium ascorbate (10 equiv) and glutathione (10 eq and 500 eq.) at 37 °C and pH 7 and the release of the IPA and DCA ligands was therefore analyzed by HPLC (Figures S15–S20). The concomitant release of both axial ligands, DCA and IPA, in the presence of 10 equiv of sodium ascorbate indicated the reduction of the Pt(IV) complexes to cisplatin(II) and oxaliplatin(II), respectively. The complexes were reduced completely after 36 h (complex **1**) and 48 h (complex **2**). By contrast, 10 equiv of glutathione was not enough to reduce the Pt(IV) complexes, and after 36 h only the starting compounds and glutathione were observed in the HPLC chromatograms. However, loss of the IPA and DCA ligands and complete reduction occurred within 24 h (complex **1**) and 30 h (complex **2**), when the glutathione concentration ratio was increased to 5 M (500 equiv). This glutathione: Pt complex ratio mimics that in cells exposed to a therapeutic concentration of cisplatin.^35−38^ Complex **2** is reduced faster than its parent dual-action prodrug [Pt(DACH)(OX)(IPA)(OH)] (80% completion after 72 h in the presence of 10 eq. ascorbate^12^ which correlates with the general order of reduction potentials for mono- and bis-carboxylated Pt(IV) complexes.^34^ By contrast, **1** is activated more slowly than the monocarboxylated complex cis,cis,trans-[Pt(NH3)2Cl2(IPA)(OH)] the reduction of which is complete after 24 h (10 eq. ascorbate).^11^ This is in line with the literature that attributes the fast reduction kinetics of Pt(IV) complexes with axial hydroxido ligands to the ability of OH to act as a bridging ligand, allowing for an inner-sphere redox mechanism.^39−41^

For *in vivo* applications and drug formulation, the stability of the prodrugs in the absence of activating reducing agents is important. The stability of **1** and **2** was assessed in freshly prepared PBS buffer/1% DMSO. The solutions were stored at 37 °C and monitored by HPLC and UV–vis spectroscopy. No changes were observed in the UV–vis spectra and chromatograms over 72 h. We also evaluated the stability of the complexes in a cell culture medium (DMEM/1% DMSO) and confirmed that the complexes are stable under cell culture conditions (Figures S11–S14). This is in marked contrast to the solution behavior of [Pt(DACH)(DCA)_2_(OX)], *cis,trans,cis*-[Pt(NH_3_)_2_(DCA)_2_Cl_2_]^39^ and cis-[Pt(1,4-DACH)(DCA)_2_Cl_2_].^16^ These complexes undergo hydrolysis within a few hours under biologically relevant conditions. On the other hand, complexes containing one axial DCA ligand and ibuprofen, 4-phenylbutyrate or valproate as the second axial ligand were found to be stable toward substitution of DCA by a hydroxido ligand.^8^ This may suggest that two electron-withdrawing haloacetate ligands are needed to activate the Pt center for nucleophilic attack.

**Figure 5:**
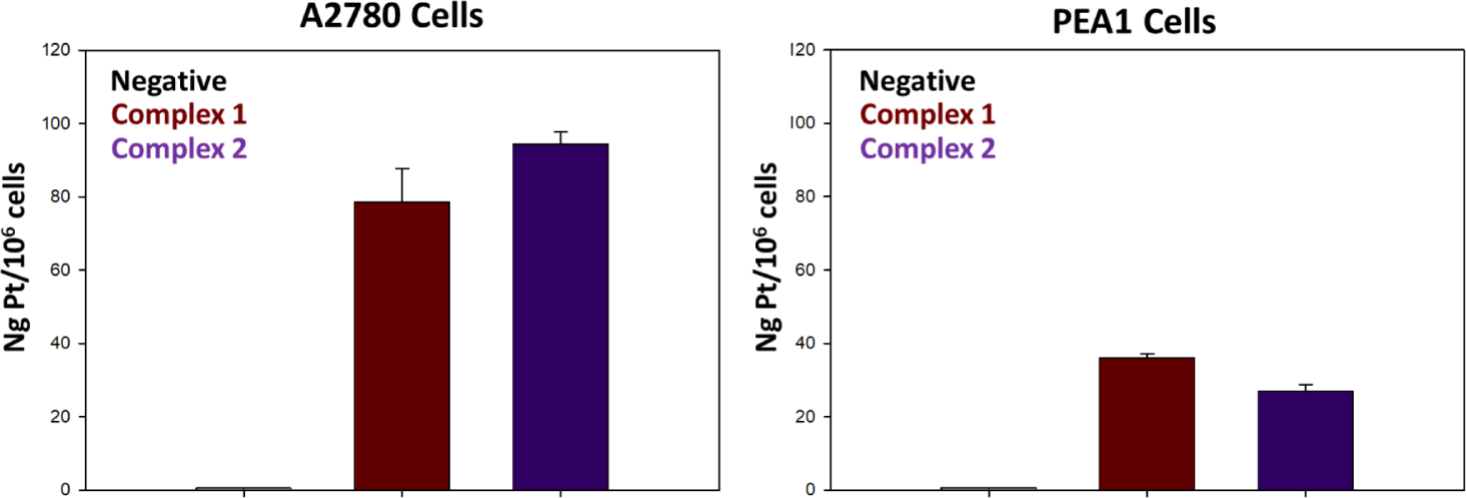
Cellular uptake of complexes **1** and **2** in A2780 and PEA1 cells incubated for 24 h with the compounds at 5 μM equimolar concentration. Negative controls refer to samples treated with vehicles only.

The lipophilicity is an important parameter for the accumulation of a drug in cancer cells. The log *P*_o/w_ values of complexes **1** and **2** were determined to be −0.911 ± 0.006 and −0.323 ± 0.003, respectively. By comparison, the parent complex of **1**, *cis,cis,trans*-[Pt(NH_3_)_2_(IPA)(OH)], has a logP_o/w_ value of −0.110 ± 0.05.^11^ The DCA ligand thus leads to an increase in the hydrophilicity. The solubility of complex **1** and complex **2** in 0.9% NaCl solution is 3.4 and 0.4 mol/L, respectively. In order to evaluate the relationship between cytotoxicity and cellular accumulation, the cellular uptake of **1** and **2** into A2780 and PEA1 cells was quantified by using ICP-MS. Figure 5 shows the intracellular Pt levels (as nanograms of metal per 10^6^ cells) after 24 h incubation with an equimolar dose of 5 μM of **1** and **2**. The Pt content was higher in A2780 cells than in PEA1 cells, in line with the higher activity of the complexes in the former cell line. In the case of the A2780 cells, the more prominent cytotoxicity of the oxaliplatin derivative compared to the cisplatin derivative correlates with the relative uptake of **1** and **2**.

**Figure 6:**
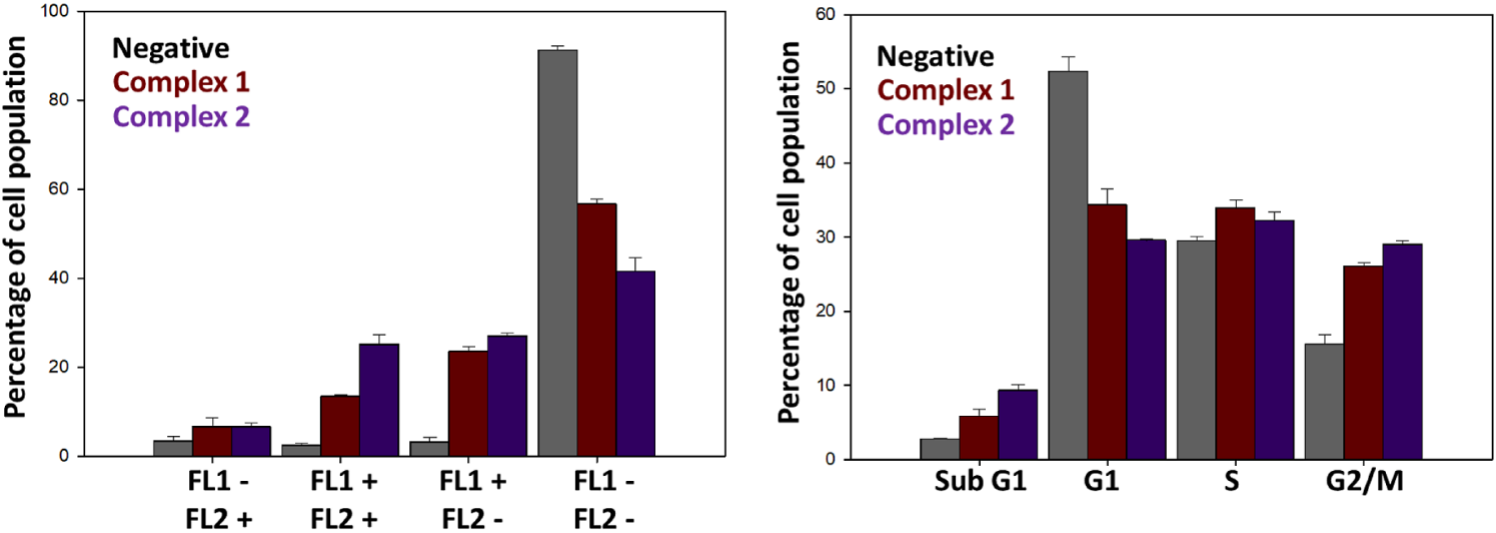
Induction of apoptosis (Left) and cell cycle investigations (Right) on A2780 ovarian cancer cells exposed to equipotent concentrations of complexes **1** and **2** (1X IC_50_). These experiments included 24 h of drug exposure and no recovery time. Untreated cells were used as negative controls.

To gain insight into the cell death pathway that is activated in treated cells, we studied the ability of complexes **1** and **2** to induce apoptosis in A2780 cells. Flow cytometry investigations of cells costained with Annexin V and propidium iodide indicate the activation of apoptosis at an early time frame, after only 24 h of drug exposure (Figure 6 and Table S3). This experiment allows for the simultaneous detection of four distinctive populations as a combination of low and high fluorescence in a green channel measuring annexin (FL1) and a red channel measuring propidium iodide (FL2). As shown in the figure, the first set of cells is nonviable and is only stained with propidium iodide (FL2+) while the second set gives a positive response in both the red and green channels (FL2+ and FL1+). These cells are considered late apoptotic and reflect a population in which there has been an exposure of the phosphatidylserine on the outer membrane and blebbing has started to occur. As expected, this subset is significantly higher in cells exposed to complexes **1** and **2**, with higher percentages of cell population for the second complex. The third population set reflects cells in early apoptosis in which the cellular membranes have not yet been compromised; therefore, only green annexin fluorescence (FL1+). The last subset, which represents viable cells, does not have considerable fluorescence in either channel (FL1– and FL2−) and is, as expected, the highest in the untreated samples.

Next, we investigated the cellular damage that could be involved in the activation of apoptotic cell death. Events that trigger apoptosis include DNA damage and oxidative stress.^42^ Pt agents are generally associated with DNA binding, and the coordination of the *cis*-Pt(NH_3_)_2_^2+^ entity to two neighboring guanine bases in DNA is believed to play a crucial role in the mode of action of cisplatin and carboplatin. The formation of DNA lesions triggers the DNA damage response (DDR). When the DDR fails, mitochondrial apoptosis is initiated.^43,44^ To allow for time to repair the DNA lesion, the DDR activates the blockage of the cell cycle at the G2/M phase.^45^ We therefore investigated the effect of the Pt(IV) prodrugs on the cell cycle distribution (Figure 6 and Table S4). In this case, staining with propidium iodide (PI) allows for the flow cytometry detection of DNA content in cancer cells and its relationship with their location within the cell cycle. For the untreated controls, the population is mainly distributed in the G1 phase as expected, with a minimum percentage in the sub-G1 phase, which is indicative of nonviable cells in which the DNA is fragmented. Cells treated with complexes **1** and **2** show a reduction of the G1 population and a marked increase in the G2/M phase. There are no statistically significant changes in the number of cells present in the S phase. This observation is consistent with reported results of other platinum drugs, with cisplatin causing G2/M arrest in ovarian cells.^46^ The extent of the arrest observed for complexes **1** and **2** is consistent with their individual potencies in the A2780 cell line, which would suggest that indeed part of the activation of apoptosis could be in response to DNA damage caused by the Pt(IV) complexes.

**Figure 7:**
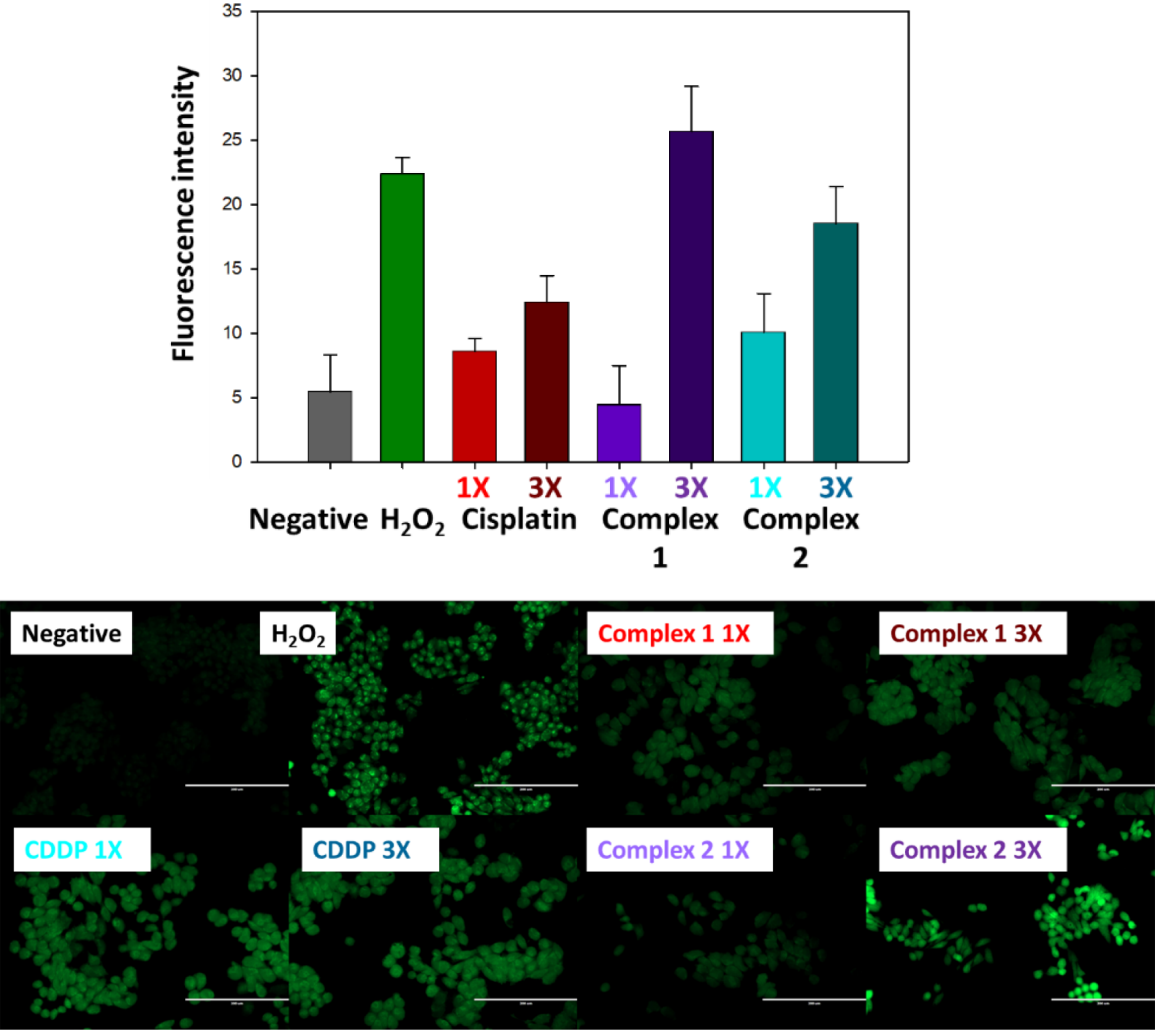
Induction of ROS in A2780 cells exposed to Pt(IV) complexes **1** and **2** at equipotent concentrations equal to 1X and 3X IC_50_ values. The fluorescence intensity (Top) and fluorescence microscopy (Bottom) experiments included a 24 h drug exposure period, no recovery time, and hydrogen peroxide as positive controls. Untreated cells were used as negative controls.

We showed previously that the IPA complexes c*is,cis,trans*-[Pt(NH_3_)_2_Cl_2_(IPA)(OH)] and *trans*-[Pt(DACH)(OX)(IPA)(OH)] induce oxidative stress in cancer cells.^11,12^ We therefore hypothesized that mixed IPA/DCA complexes **1** and **2** also increase cellular ROS levels, which could also be consistent with the induction of apoptosis observed. This could occur in addition to the DNA damage observed and would reinforce the idea of the Pt(IV) drugs being multitargeted. This would be particularly relevant in a clinical setting taking into consideration that there is agreement of a strong link between the levels of oxidative stress and the progression and chemosensitivity of ovarian cancer.^47^Figure 7 (and Table S5) shows the concentration-dependent ROS generation in cells exposed to **1** or **2**. In these experiments, we used cell-permeant 2′,7′-dichlorodihydrofluorescein diacetate (H_2_DCFDA). This chemical is readily taken up by live cells and, upon reaction with ROS emits green fluorescence that can be read at 530 nm. Mammalian cells use low levels of ROS for signaling purposes, and therefore, untreated controls show low green fluorescence. Cells treated with complex **2** have higher ROS levels than cells treated with complex **1** or cisplatin, with the effect being concentration dependent. As expected, cells treated with hydrogen peroxide as a positive control had high intensity.

**Figure 8:**
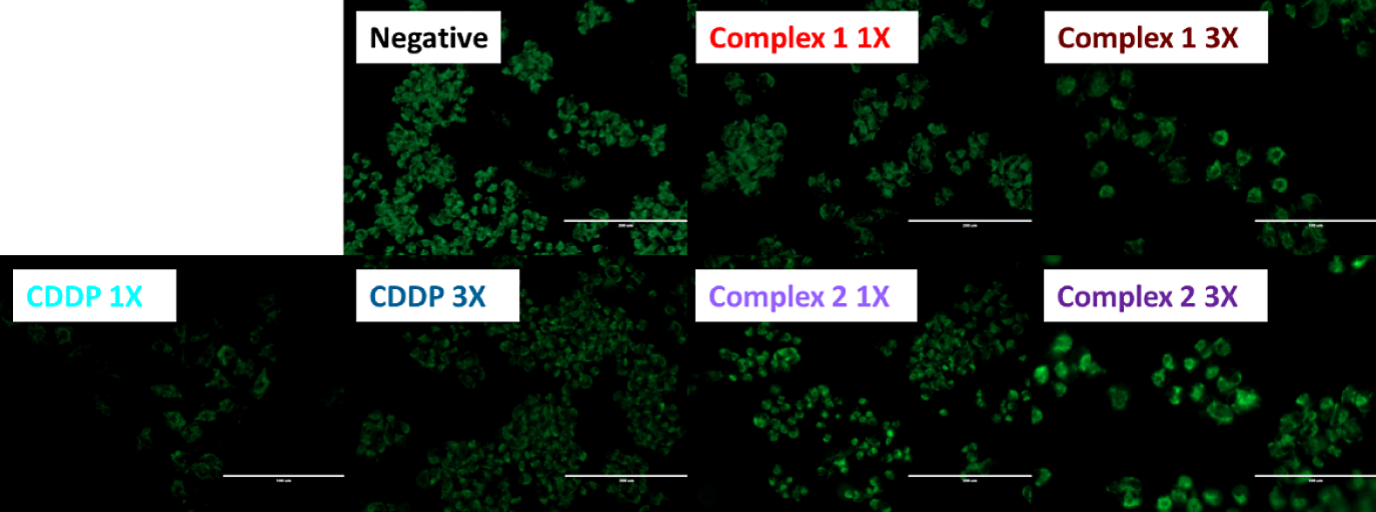
Mitochondrial function in A2780 cells exposed to Pt(IV) complexes **1** and **2** at equipotent concentrations equal to 1X and 3X IC_50_ values. Fluorescence microscopy experiments included a 24 h drug exposure period and no recovery time.

Although Pt(II) complexes in the clinic are usually categorized as DNA damaging agents, their effect on mitochondrial function has been intensely investigated, particularly paying attention to the role that ROS generation has in modifications of mitochondrial content.^48^ This link has also been established for other metal-based multitargeted complexes with Ru(II) and Os(II) centers, in which the cytotoxicity of the complexes directly correlates to their ability to generate ROS and the observed reduction of mitochondrial function.^49,50^ Therefore, given the ROS induction caused by complexes **1** and **2**, we considered it important to further evaluate their impact on mitochondrial activity. In this case, we used Rhodamine-123 as a cell-permeant, cationic dye to report active mitochondria. Figure 8 reveals that under the conditions of the experiment, there is no significant disruption caused by complexes **1** and **2**, The pictures do show that there is a slight reduction of mitochondrial function after cisplatin treatment, as expected. This observation should be further investigated to discard a time frame discrepancy between the disruption and the drug exposure time. Links between the generation of ROS and mitochondrial dysfunction have also been reported for cells exposed to oxaliplatin. There is evidence which connects the oxidative stress caused by the platinum drug to an inhibition of the Nrf2 signaling pathway and subsequent activation of ferroptosis,^51^ as well as, evidence of chemosensitation via the JNK and p38 MAPK pathways ahead of apoptosis when oxaliplatin is administered with ROS inducers. The latter could relate to the mechanism of complex 2 considering the possibility of oxaliplatin release, as well as, ROS induction caused by the metal center.^52^ Remarkably, the connection between mt-ROS induction mitochondrial function and chemosensitivity may even be the basis for biomarkers for platinum response.^48^

## Conclusions

A new promising Pt(IV) complex for the treatment of platinum-resistant ovarian carcinomas has been identified. The Pt(IV) derivative of oxaliplatin, *trans*-[Pt(DACH)(OX)(IPA)(DCA)] (**2**), is a strong cytotoxin in various ovarian cancer cell lines and maintains its low micro- to submicromolar IC_50_ values in cisplatin-resistant PEA2 and A2780cis cells.

Mechanistic studies indicate a multifactorial mechanism that involves ROS generation and possible DNA damage evidenced by cell cycle arrest. The relative cytotoxicities of **2** and the analogous Pt(IV) derivative of cisplatin correlate with the ability to increase cellular ROS levels. It is notable that the trend in activity, **2** > **1**, is the opposite of that of the parent complexes that contain the IPA ligand only (c*is,cis,trans*-[Pt(NH_3_)_2_Cl_2_(IPA)(OH)] > *trans*-[Pt(DACH)(OX)(IPA)(OH)]). In fact, the addition of the second bioactive ligand DCA significantly enhances the potency of the oxaliplatin-based complex. DCA alone is active at concentrations in the millimolar range only^53^ which might be due to poor cellular uptake of its deprotonated form that is present at physiological pH value. Coordination to the Pt scaffold can facilitate cellular accumulation of anionic DCA. In summary, this study shows that Pt(IV) prodrugs are a promising avenue in the fight against recurrent cisplatin-resistant ovarian cancers.

## Supporting Information Available

The synthetic scheme followed for the generation of complexes **1** and **2** (Scheme S1), as well as, their physicochemical characterization by NMR, MS, and IR (Figures S1–S10). The data corresponding to investigations of chemical stability in biologically relevant matrixes and the activation by reduction of the complexes can be found in Figures S11–S19. Numerical data for biological experiments is included in Tables S1–S5 (PDF)
