# Supplementary material for: Multiaction Pt(IV) Complexes: Cytotoxicity in Ovarian Cancer Cell Lines and Mechanistic Studies
Source: Inorg Chem. 2024 Jul 31;63(32):14958–68. doi: 10.1021/acs.inorgchem.4c01586 (PMC11323244; doi:10.1021/acs.inorgchem.4c01586)
Supplement: Supplementary file 1 — ic4c01586_si_001.pdf [file ic4c01586_si_001.pdf]

Multi-action Pt(IV) complexes: Cytotoxicity in ovarian cancer cell lines and mechanistic studies

Leila Tabrizi[1,5], Alan M. Jones[2], Isolda Romero-Canelon*[2,3] and Andrea Erxleben*[1,4]

[1] School of Biological and Chemical Sciences, University of Galway, Galway, Ireland, H91 TK33
[2] School of Pharmacy, University of Birmingham, Birmingham UK, B15 2TT
[3] Department of Chemistry, University of Warwick, Coventry UK, CV4 7AL
[4] Synthesis and Solid State Pharmaceutical Centre (SSPC), Limerick, Ireland, V94 T9PX
[5] School of Chemical Sciences, Dublin City University, Dublin, Ireland, D09 W6Y4

Supporting Information

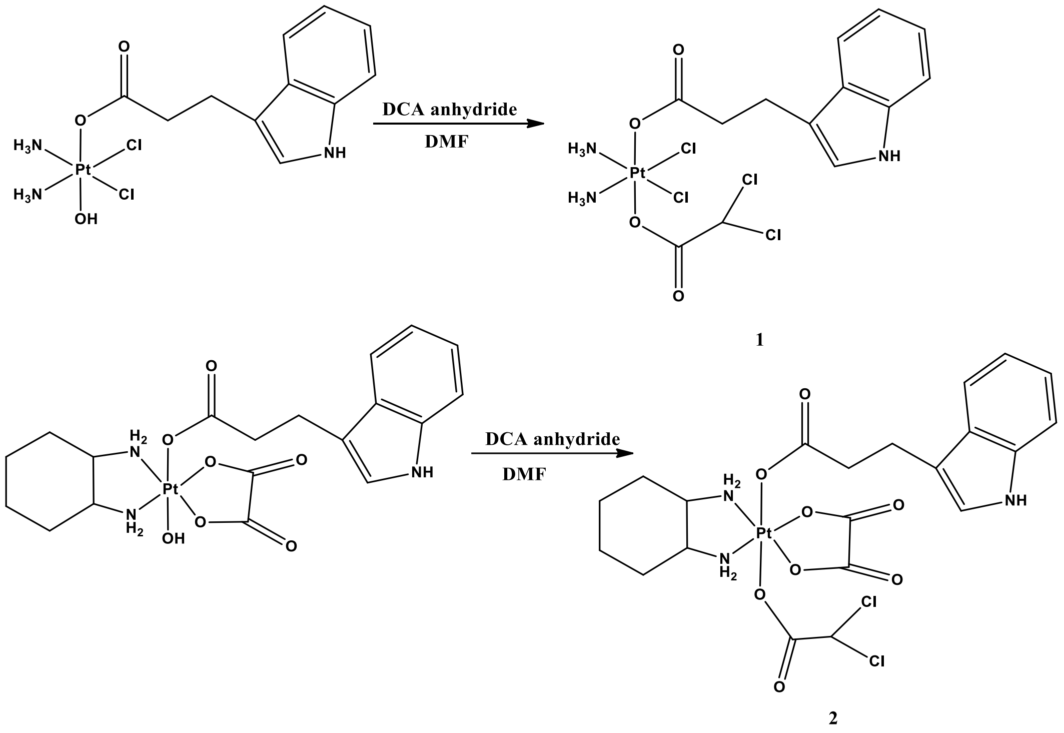

Scheme S1. Synthesis of complexes **1** and **2**.

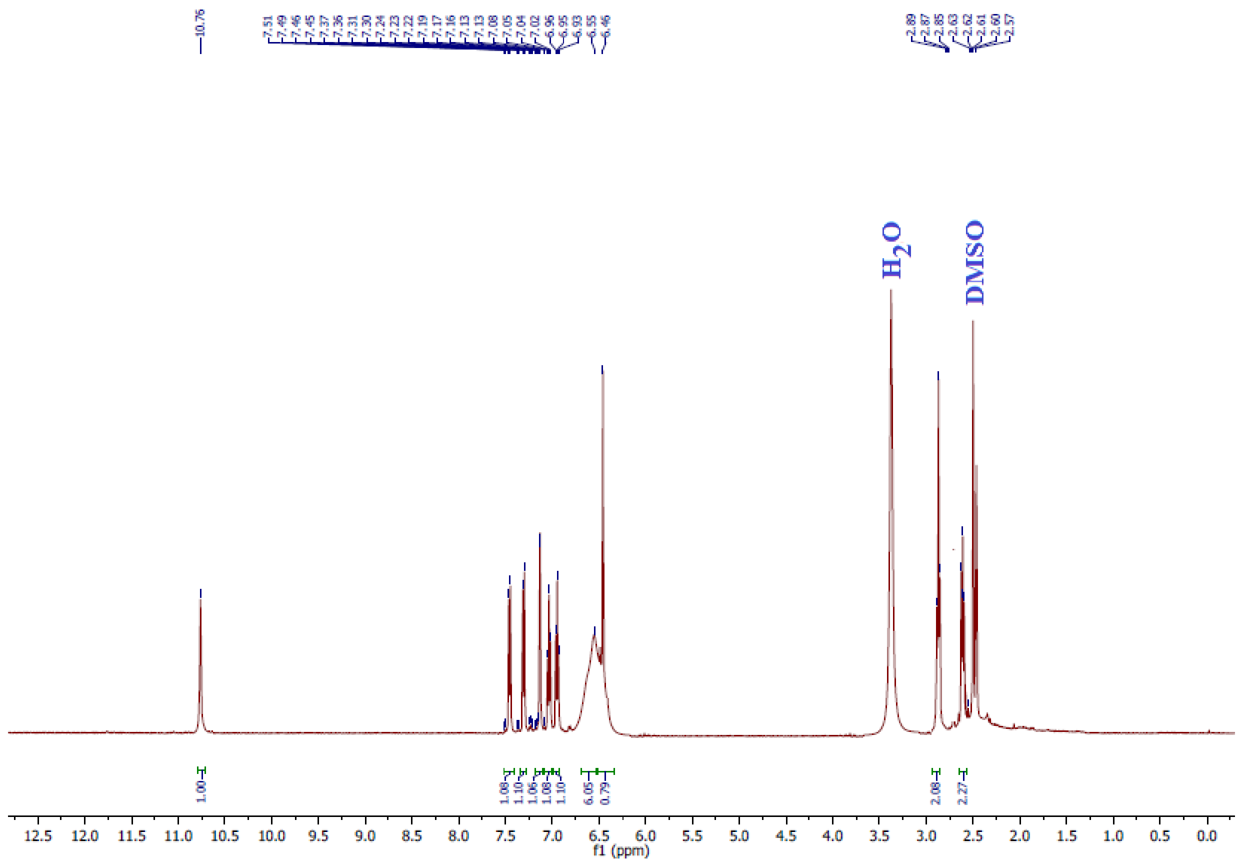

Figure S1. ^{1}H NMR spectrum of complex **1** (DMSO-d_6).

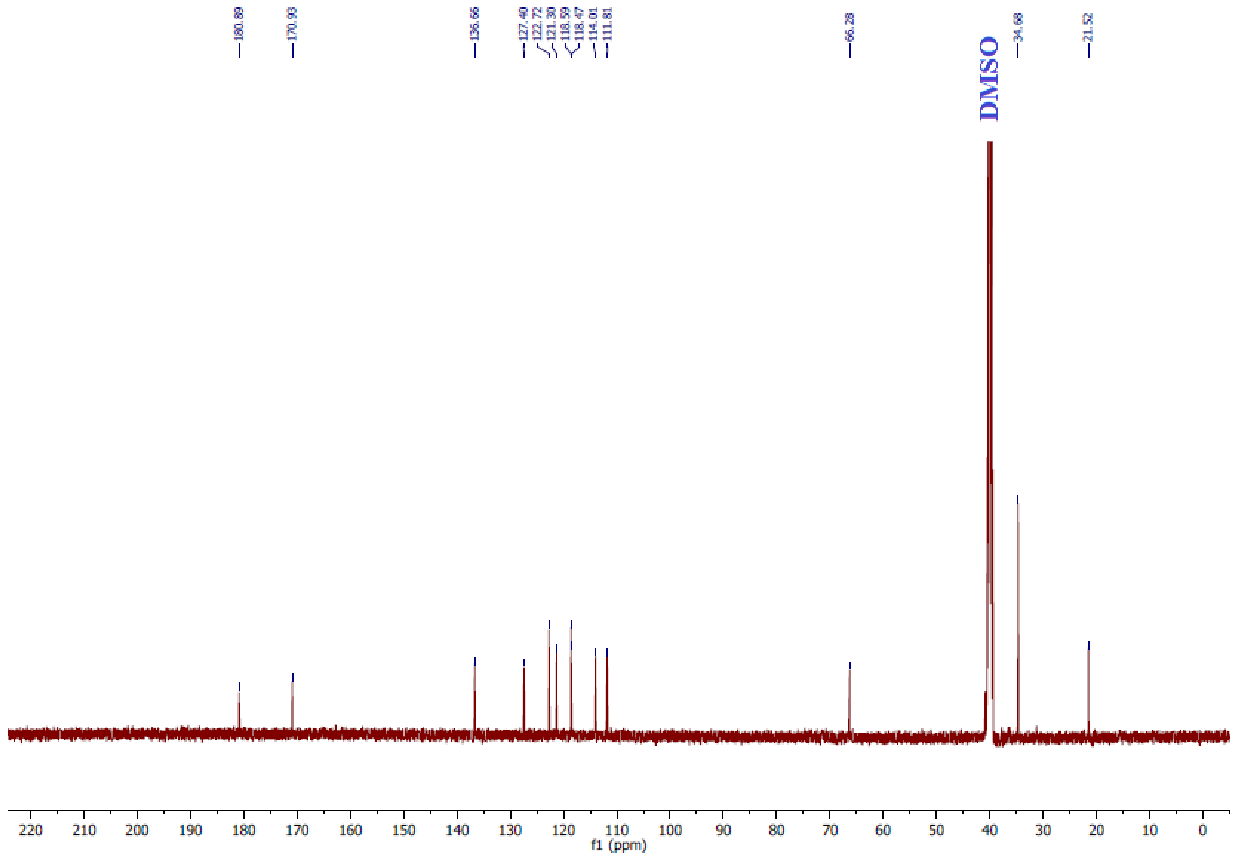

Figure S2. ^{13}C NMR spectrum of complex **1** (DMSO-d_6).

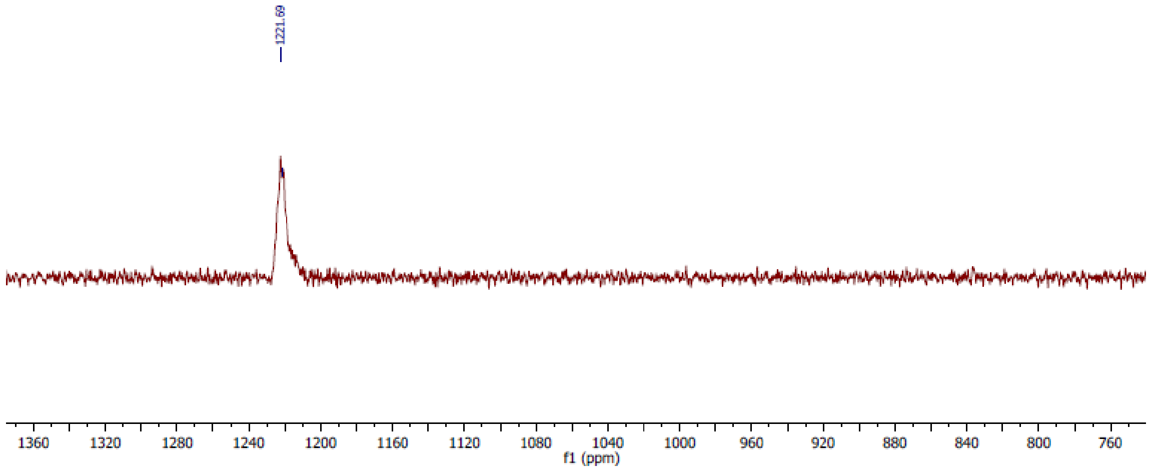

Figure S3. ^{195}Pt NMR spectrum of complex **1** (DMF/D_2O).

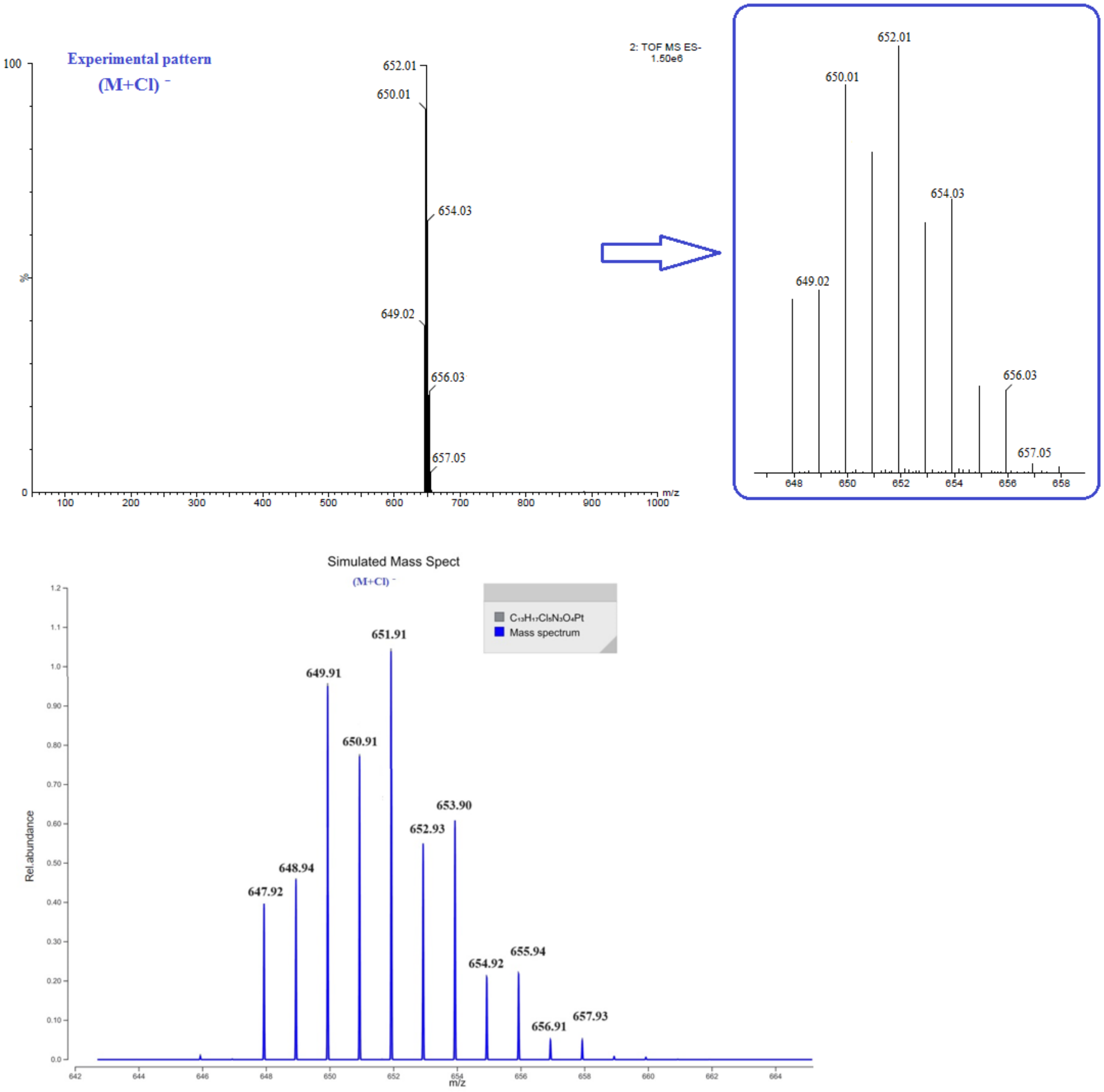

Figure S4. ESI-MS of complex **1** (in acetonitrile).

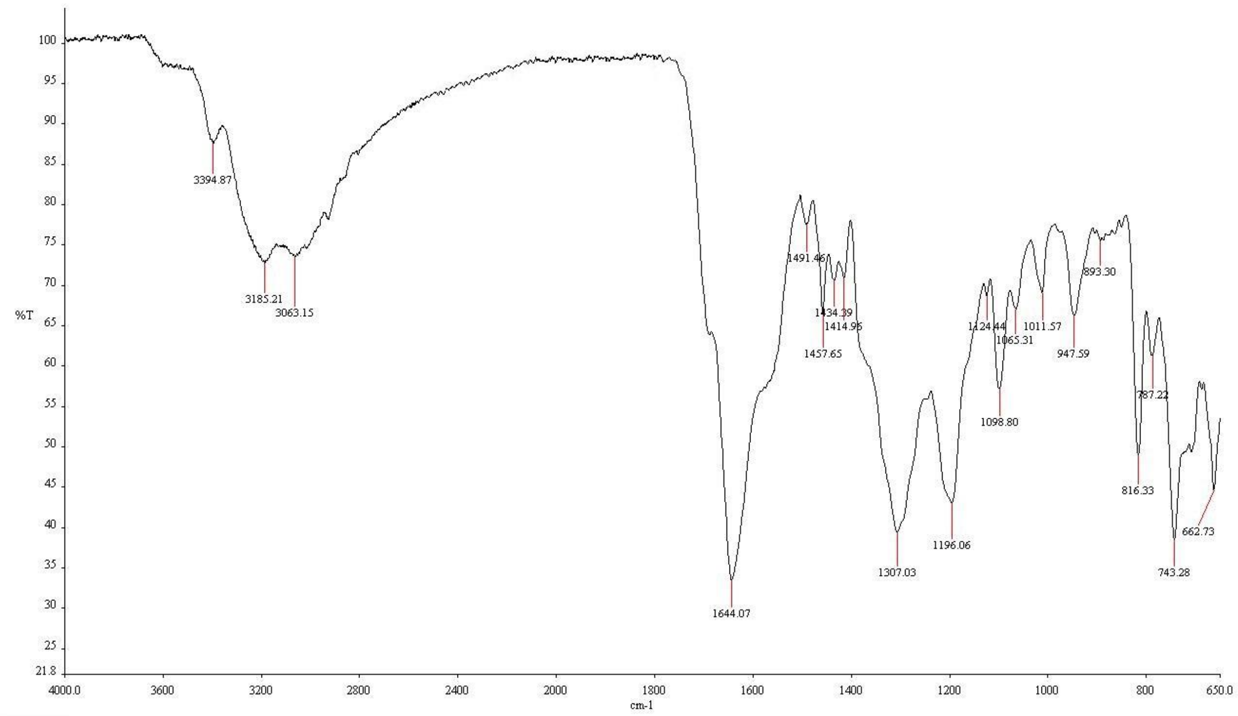

Figure S5. IR spectrum of complex **1**.

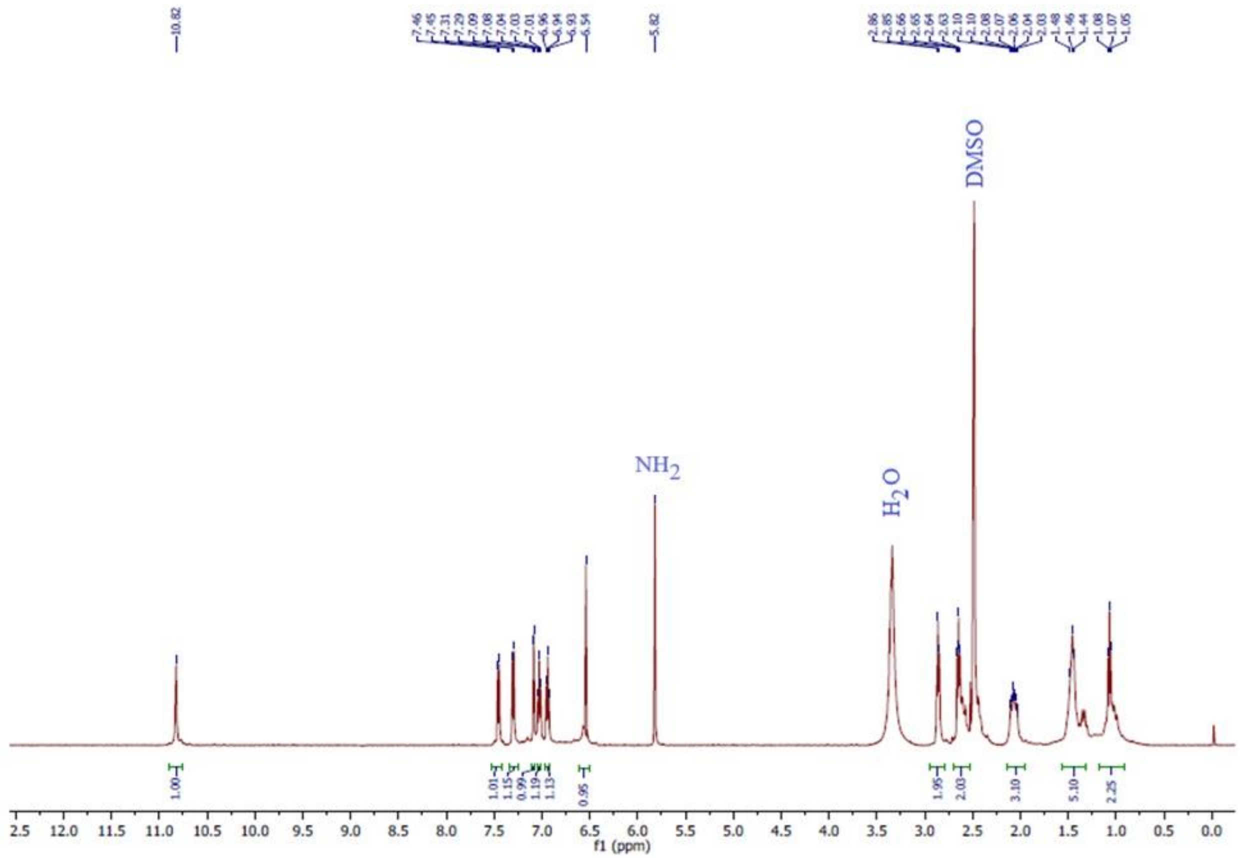

Figure S6. ^1H NMR spectrum of complex **2** (DMSO-d_6).

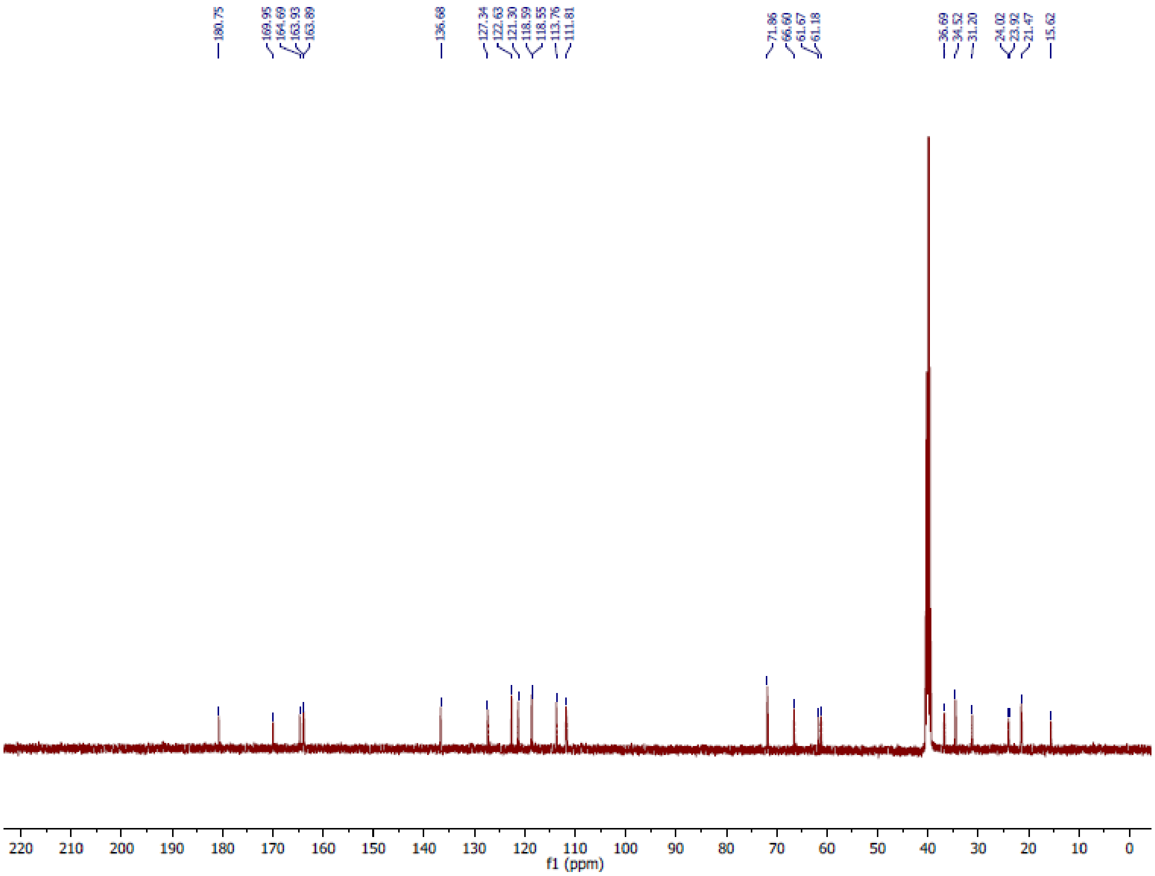

Figure S7. ^{13}C NMR spectrum of complex **2** (DMSO-d_6).

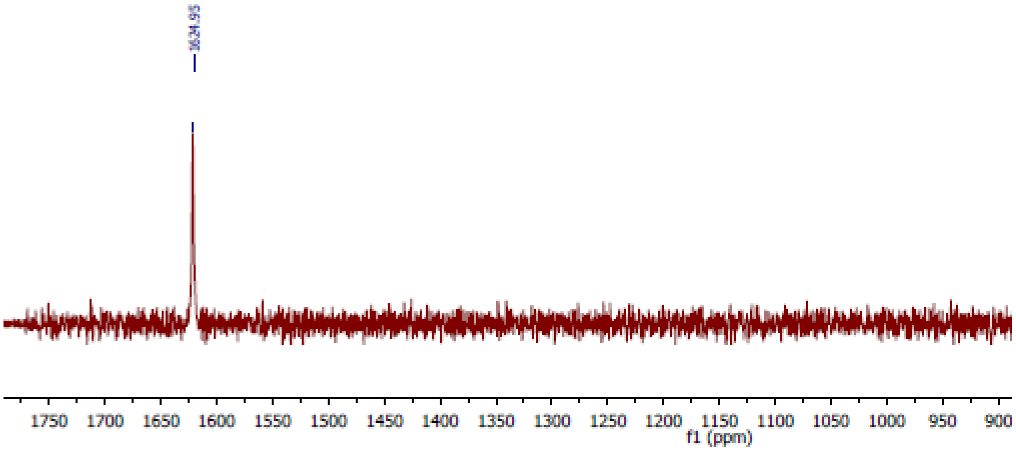

Figure S8. ^{195}Pt NMR spectrum of complex **2** (DMF/D_2O).

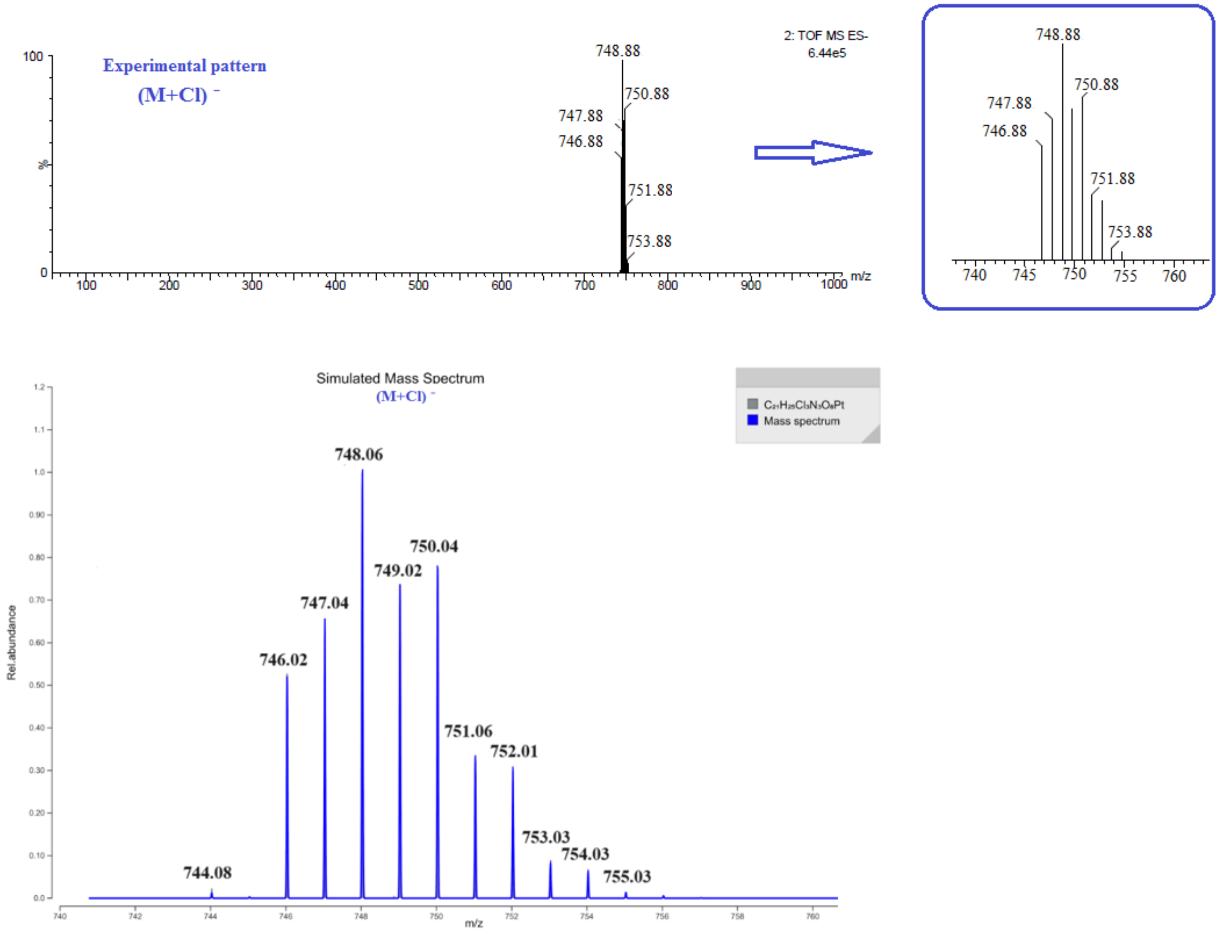

Figure S9. ESI-MS of complex **2** (in acetonitrile).

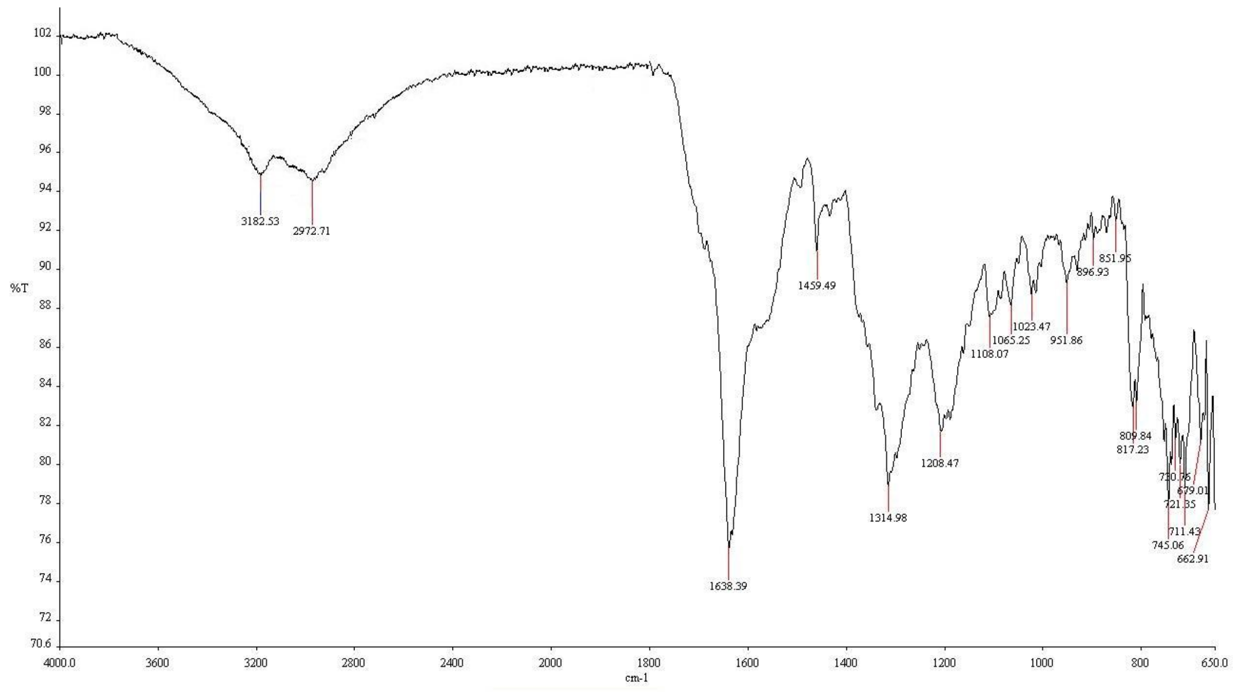

Figure S10. IR spectrum of complex **2**.

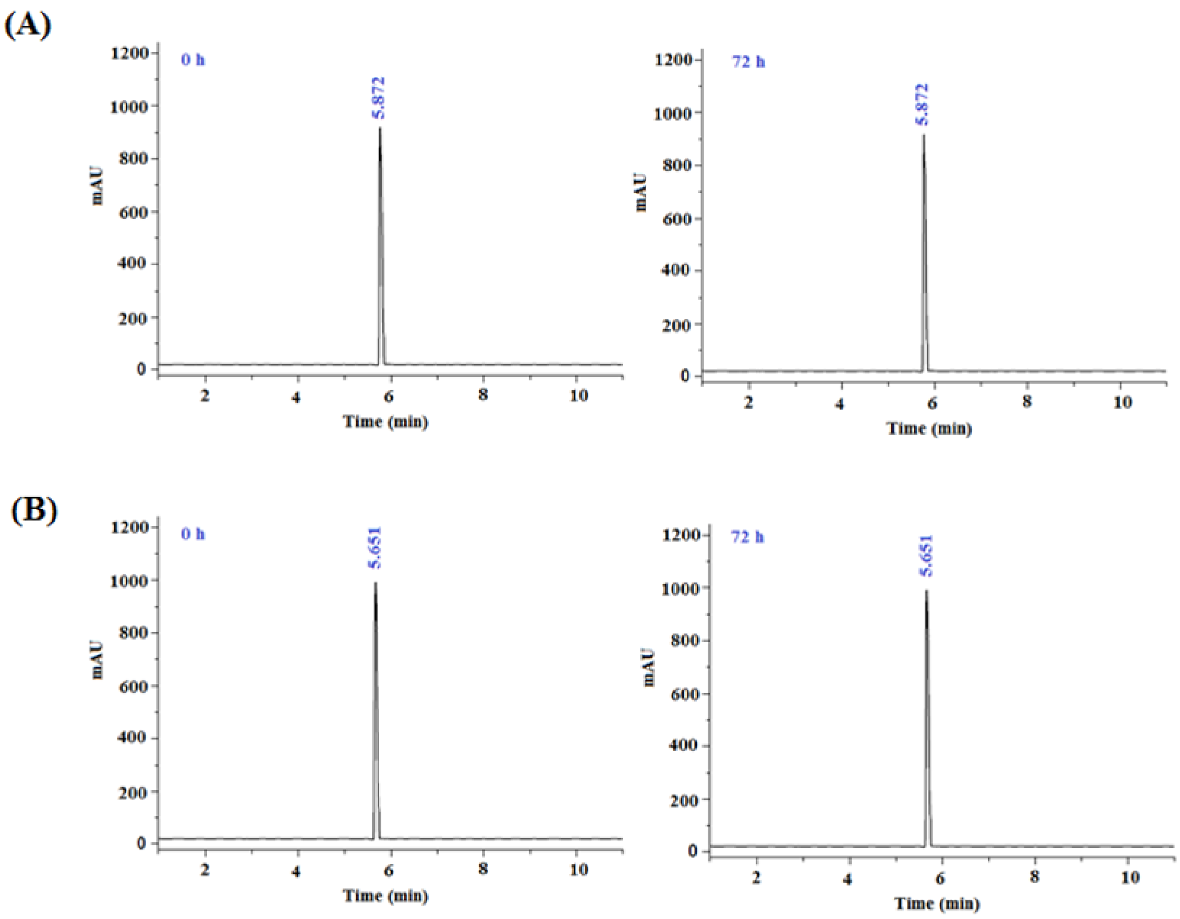

Figure S11. (A) HPLC chromatogram of complex **1** (1×10^{-3} M) recorded immediately after dissolution in DMEM (Dulbecco's Modified Eagle's Medium - high glucose) /1% DMSO solution (0 h) and after 72 h incubation at 37 ˚C; mobile phase: 50:50 methanol (0.1% trifluoroacetic acid) : water (0.1% trifluoroacetic acid). (B) HPLC chromatogram of complex **1** (1×10^{-3} M) recorded immediately after dissolution in freshly prepared PBS buffer/1% DMSO solution (0 h) and after 72 h incubation at 37 ˚C; mobile phase: 50:50 methanol (0.1% trifluoroacetic acid): water (0.1% trifluoroacetic acid).

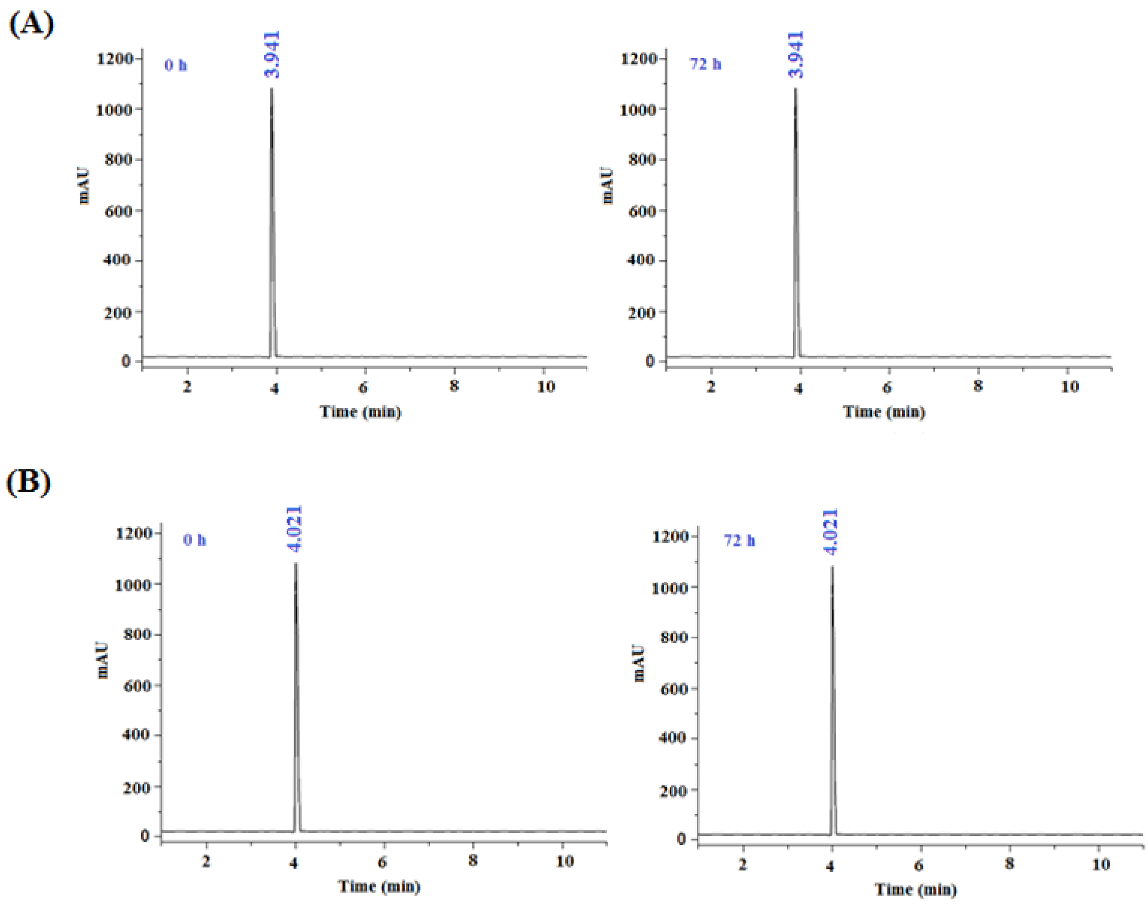

Figure S12. (A) HPLC chromatogram of complex **2** (1 × 10^{-3} M) immediately after dissolution in DMEM (Dulbecco's Modified Eagle's Medium - high glucose) /1% DMSO solution (0 h) and after 72 h incubation at 37 ˚C; mobile phase: 50:50 methanol (0.1% trifluoroacetic acid) : water (0.1% trifluoroacetic acid). (B) HPLC chromatogram of complex **2** (1 × 10^{-3} M) immediately after dissolution in freshly prepared PBS buffer/1% DMSO solution (0 h) and after 72 h incubation at 37 ˚C; mobile phase: 70:30 acetonitrile (0.1% trifluoroacetic acid) : water (0.1% trifluoroacetic acid).

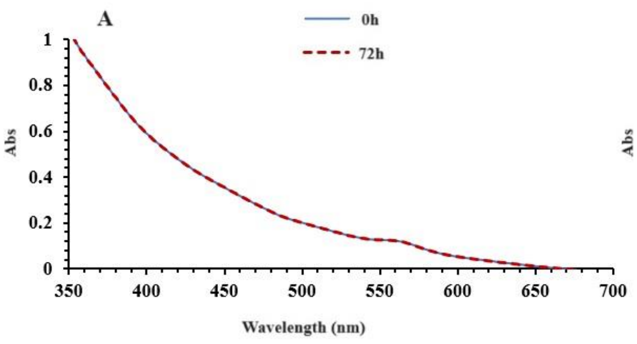

Figure S13. (A) UV-Vis spectrum of complex **1** (1×10^{-3} M) immediately after dissolution in DMEM (Dulbecco's Modified Eagle's Medium - high glucose) /1% DMSO solution (0 h) and after 72 h incubation at 37 ˚C. (B) UV-vis spectrum of complex **1** (1×10^{-3} M) immediately after dissolution in freshly prepared PBS buffer/1% DMSO solution (0 h) and after 72 h incubation at 37 ˚C.

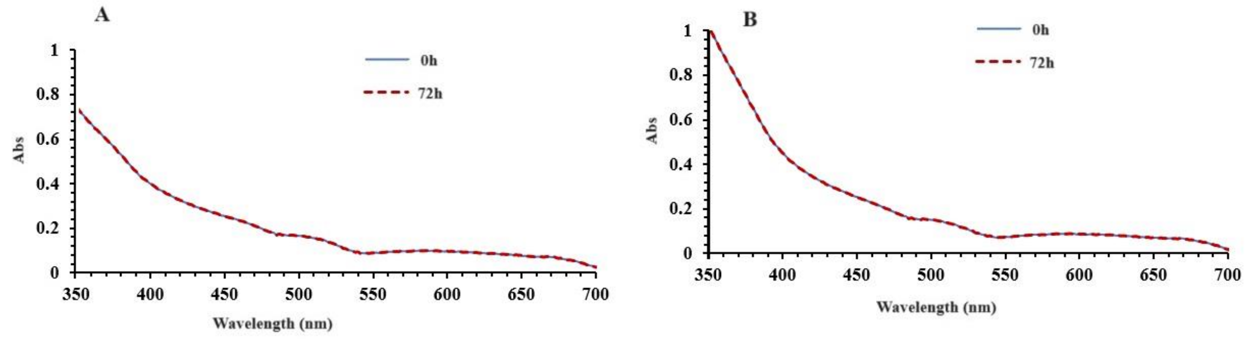

Figure S14. (A) UV-vis spectrum of complex **2** (1×10^{-3} M) immediately after dissolution in DMEM (Dulbecco's Modified Eagle's Medium - high glucose) /1% DMSO solution (0 h) and after 72 h incubation at 37 ˚C. (B) UV-vis spectrum of complex **2** (1×10^{-3} M) immediately after dissolution in freshly prepared PBS buffer/1% DMSO solution (0 h) and after 72 h incubation at 37 ˚C.

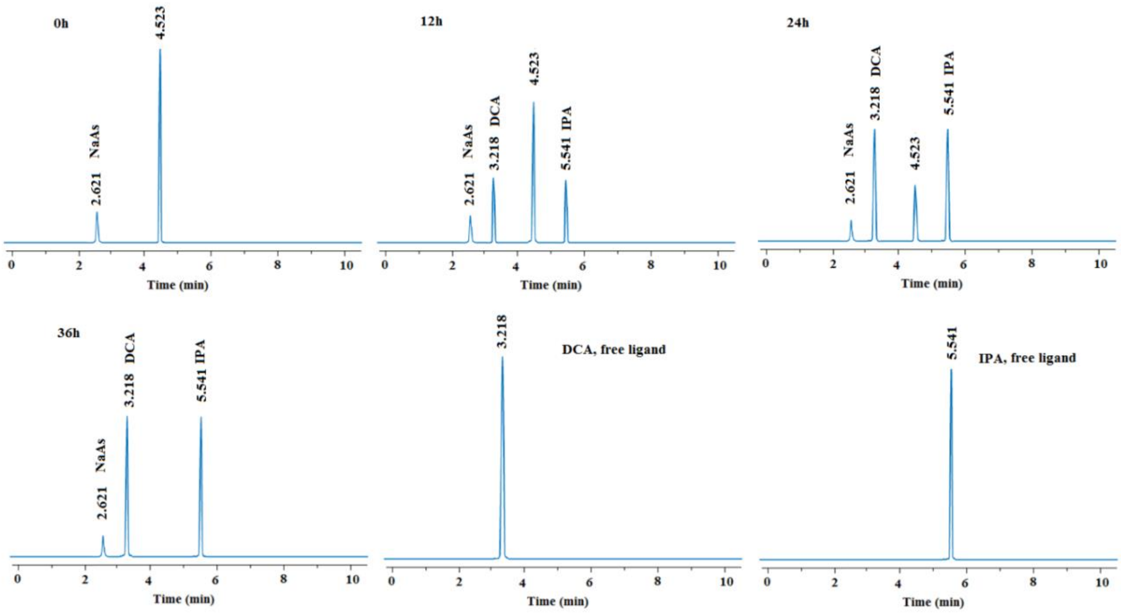

Figure S15. HPLC chromatograms of the reaction of complex **1** with 10 eq sodium ascorbate (NaAs) at 37 °C and pH 7; mobile phase: 70:30 acetonitrile (0.1% trifluoroacetic acid) : water (0.1% trifluoroacetic acid). The chromatograms of the free ligands are shown for comparison purposes.

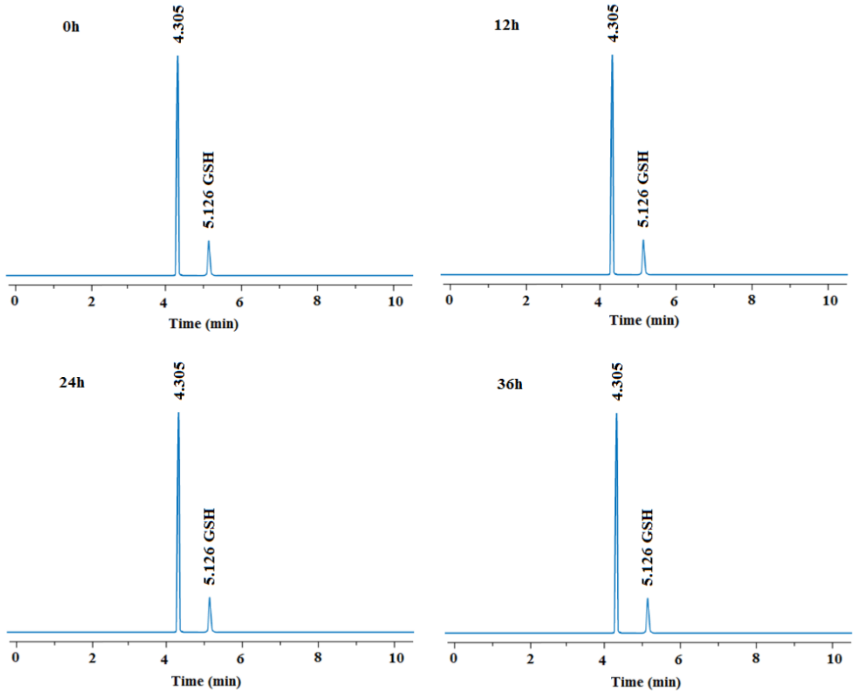

Figure S16. HPLC chromatograms of the reaction of complex **1** with 10 eq glutathione at 37 °C and pH 7; mobile phase: 70:30 acetonitrile (0.1% trifluoroacetic acid): water (0.1% trifluoroacetic acid).

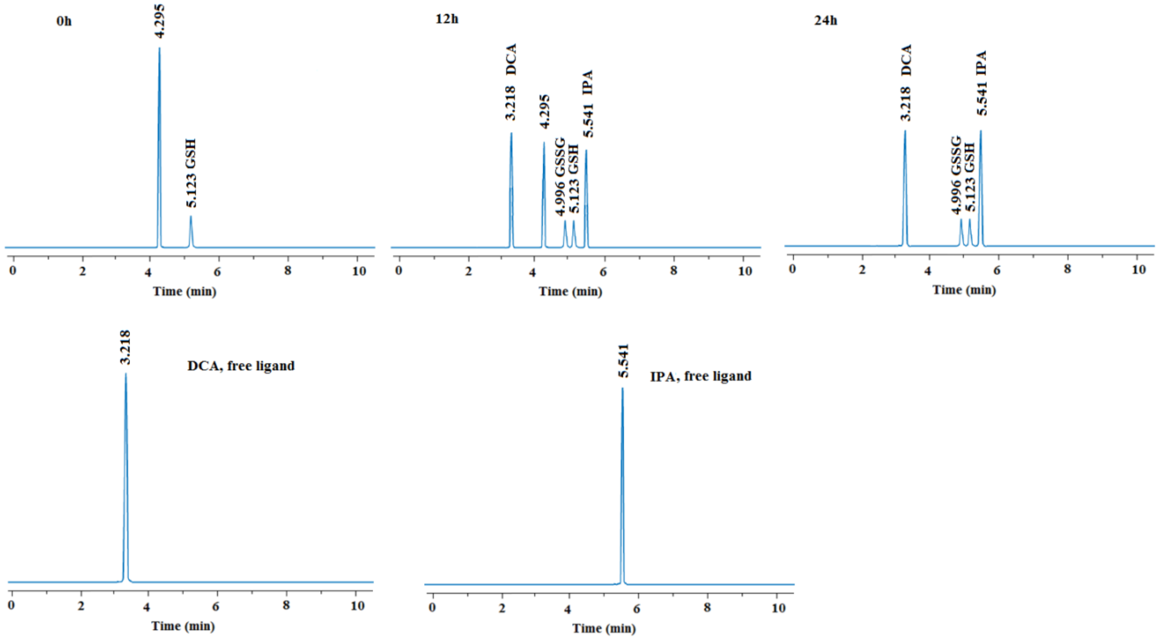

Figure S17. HPLC chromatograms of the reaction of complex **1** with 500 eq glutathione at 37 °C and pH 7; mobile phase 70:30 acetonitrile (0.1% trifluoroacetic acid): water (0.1% trifluoroacetic acid).

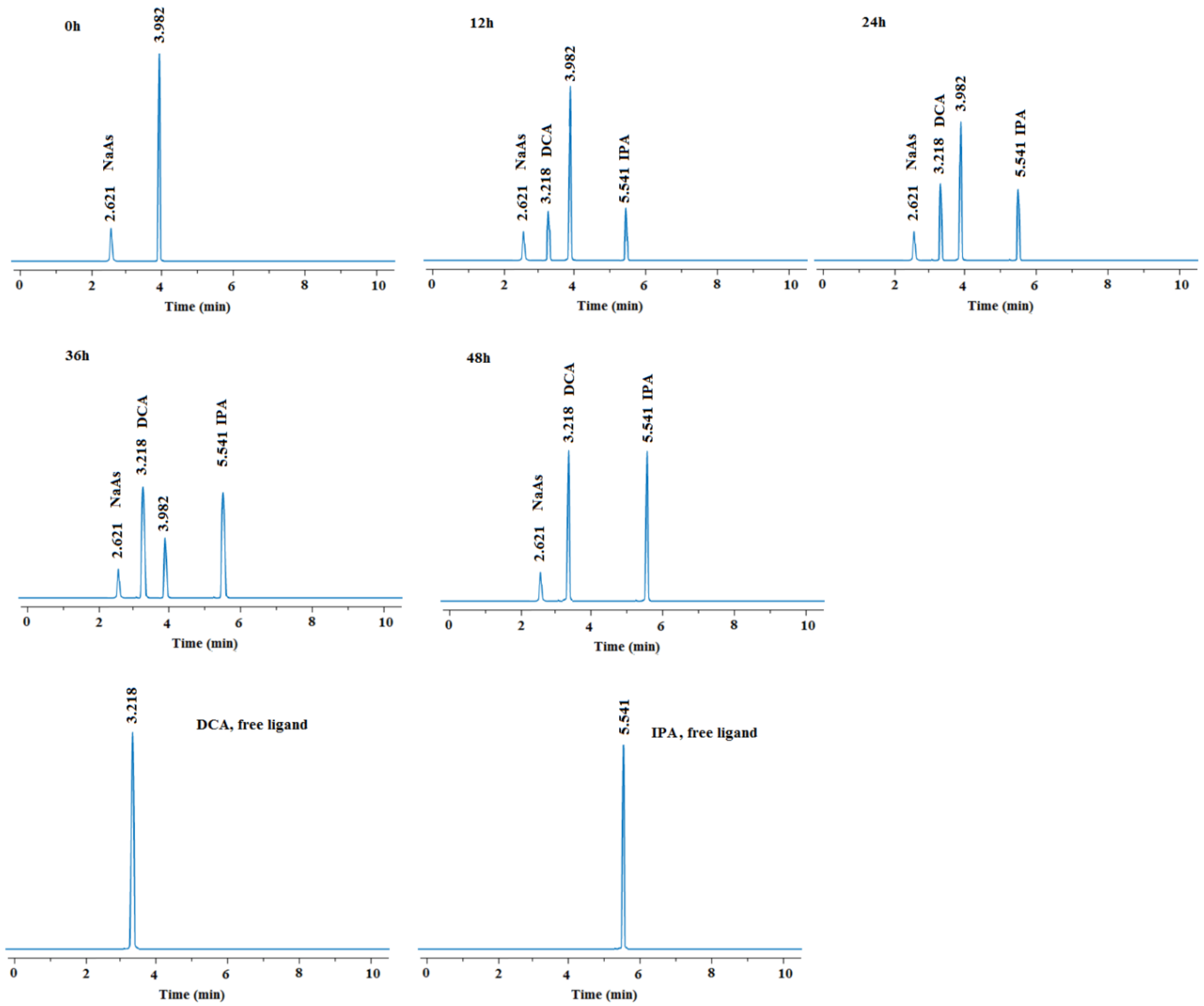

Figure S18. HPLC chromatograms of the reaction of complex **2** with 10 eq sodium ascorbate (NaAs) at 37 °C and pH 7; mobile phase: 70:30 acetonitrile (0.1% trifluoroacetic acid): water (0.1% trifluoroacetic acid). The chromatograms of the free ligands are shown for comparison purposes.

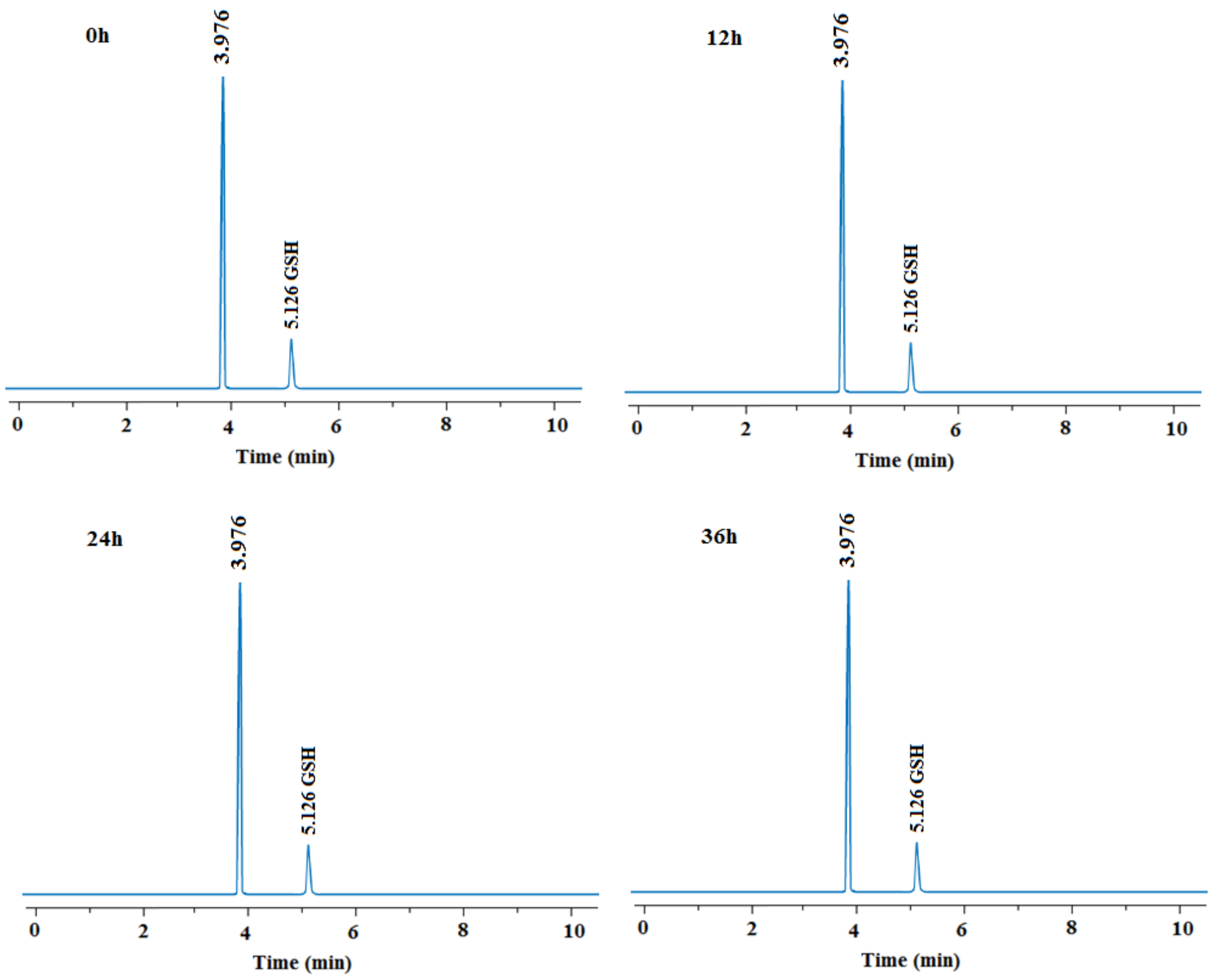

Figure S19. HPLC chromatograms of the reaction of complex **2** with 10 eq glutathione at 37 °C and pH 7; mobile phase: 70:30 acetonitrile (0.1% trifluoroacetic acid): water (0.1% trifluoroacetic acid).

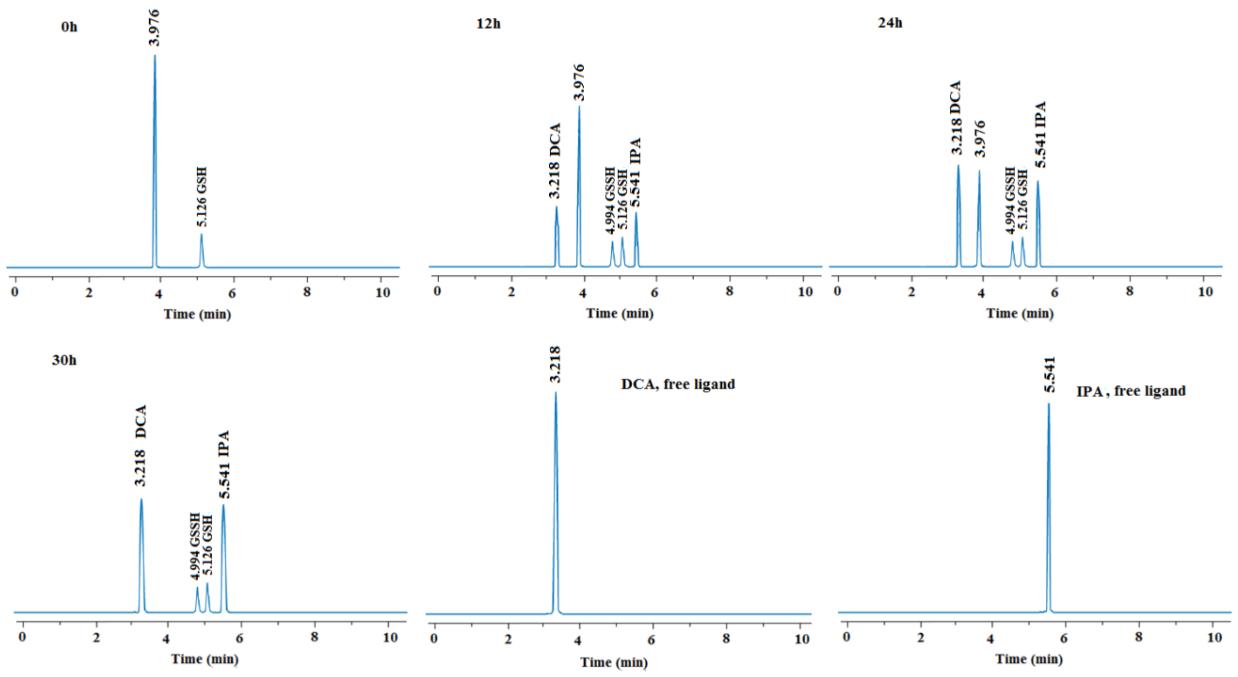

Figure S20. HPLC chromatograms of the reaction of complex **2** with 500 eq glutathione at 37 °C and pH 7; mobile phase: 70:30 acetonitrile (0.1% trifluoroacetic acid): water (0.1% trifluoroacetic acid).

Table S1. Measurements of spheroid diameter. Spheroids were generated using A2780 ovarian cancer cells grown in cell repellent 96-well plates. The experiments included 24 h of drug exposure time.

	Negative controls	Cisplatin 1X	Cisplatin 3X	Complex **2** 1X	Complex **2** 3X
	772.84	628	618	703	673
	843.06	596	661	639	620
	783.81	643	603	638	652
	765.51	590	594	663	704
	841.1	636	627	691	670
	806.34	568	582	701	684
	824.62	647	602	704	701
	835.54	622	584	640	634
	841.87	643	585	686	635
	841.17	599	626	682	685
Average value	816.7	617.2	608.2	674.7	665.8
Standard deviation	30.9	27.1	25.0	27.4	29.2
Normalized to controls		0.76	0.75	0.83	0.82
Error associated		0.05	0.06	0.07	0.07

Table S2. Wound healing assay carried out using A2780 ovarian cancer cells. Wounds were inflicted and then measured after 24 h of drug exposure and no drug recovery time.

	Negative controls	Complex 1	Complex 2
	205.88	261.63	281.95
	209.01	231.93	260.16
	204.89	250.42	268.23
	195.88	260.93	263.64
	190.41	291.33	269.26
	199.9	269.62	292.85
	159.93	275.27	293.61
	172.24	255.31	289.87
	197.63	263.25	286.11
	186.2	250.99	279.63
Average value	192.1	261.0	278.5
Standard deviation	15.6	15.9	12.3

Table S3. Induction of apoptosis on A2780 ovarian cancer cells exposed to equipotent concentrations of complexes **1** and **2** equal to 3X IC_{50} values. These experiments included 24 h of drug exposure and no recovery time. Untreated cells were used as negative controls.

	Negative controls		Complex 1		Complex 2	
	Value	STD	Value	STD	Value	STD
Sub G1 Phase	2.75	0.1	5.84	0.89	9.28	0.82
G1 phase	52.29	2	34.27	2.24	29.55	0.18
S Phase	29.44	0.67	33.84	1.08	32.21	1.22
G2/M Phase	15.52	1.29	26.05	0.44	28.96	0.52

Table S4. Cell cycle analysis on A2780 ovarian cancer cells exposed to equipotent concentrations of complexes **1** and **2** equal to 3X IC_{50} values. These experiments included 24 h of drug exposure and no recovery time. Untreated cells were used as negative controls.

	Negative controls		Complex 1		Complex 2	
	Value	STD	Value	Value	STD	Value
Q1: FL1-/FL2+ Non-viable	3.31	1.08	6.56	2.03	6.54	0.88
Q2: FL1+/FL2+ Late apoptosis	2.38	0.6	13.37	0.29	25.05	2.29
Q3: FL1+/FL2- Early apoptosis	3.16	1.18	23.46	1.2	26.97	0.61
Q4: FL1-/FL2- Viable	91.15	1.02	56.61	1.18	41.44	3.09

Table S5. Induction of ROS on A2780 ovarian cancer cells exposed to equipotent concentrations of complexes **1** and **2** equal to 3X IC_{50} values, as well as, cisplatin. These experiments included 24 h of drug exposure and no recovery time. Untreated cells were used as negative controls and cells treated with hydrogen peroxide were positive controls.

	Value	STD
Negative untreated controls	5.465693	2.85
Positive controls Hydrogen peroxide	22.34984	1.28
Complex **1** 1X	8.595481	0.98
Complex **1** 3X	12.39113	2.08
Complex **2** 1X	4.446176	3.014
Complex **2** 3X	25.65321	3.49
Cisplatin 1X	10.0608	3.01
Cisplatin 3X	18.5172	2.86
